# *Plasmodium falciparum* Genomic Surveillance Reveals a Diversity of Kelch 13 Mutations in Zambia

**DOI:** 10.4269/ajtmh.25-0110

**Published:** 2025-07-31

**Authors:** Andrés Aranda-Díaz, Sydney Mwanza, Takalani I. Makhanthisa, Sonja B. Lauterbach, Faith De Amaral, Mukosha Chisenga, Brighton Mangena, Isobel Routledge, Blaženka Letinić, Bertha Kasonde, Gershom Chongwe, Mulenga C. Mwenda, John M. Miller, Tricia Hibwato, Chirwa Jacob, Busiku Hamainza, Stephen Bwalya, Japhet Chiwaula, Japhet Matoba, Chadwick Sikaala, John Chimumbwa, Amy Wesolowski, Jennifer L. Smith, Jaishree Raman, Moonga Hawela

**Affiliations:** ^1^ Malaria Elimination Initiative, Institute of Global Health Sciences, University of California, San Francisco, California, USA;; ^2^ EPPIcenter Research Program, Department of Medicine, University of California, San Francisco, California, USA;; ^3^ ISGlobal, Barcelona, Spain;; ^4^ National Health Research and Training Institute, Ndola, Zambia;; ^5^ Laboratory for Antimalarial Resistance Monitoring and Malaria Operational Research, Centre of Emerging Zoonotic and Parasitic Diseases, National Institute for Communicable Diseases, Johannesburg, South Africa;; ^6^ SADC Malaria Elimination Eight Secretariat, Windhoek, Namibia;; ^7^ PATH-Malaria Control and Elimination Partnership in Africa, Lusaka, Zambia;; ^8^ National Malaria Elimination Centre, Ministry of Health, Lusaka, Zambia;; ^9^ Department of Epidemiology, Johns Hopkins University Bloomberg School of Public Health, Baltimore, Maryland, USA;; ^10^ Department of Epidemiology & Biostatistics, University of California, San Francisco, California, USA;; ^11^ Wits Research Institute for Malaria, University of Witwatersrand, Johannesburg, South Africa;; ^12^ University of Pretoria Institute for Sustainable Malaria Control, University of Pretoria, Pretoria, South Africa

## Abstract

Antimalarials are central to Zambia’s strategies for malaria control and elimination. Antimalarial drug resistance poses a significant threat to the effectiveness of artemisinin-based combination therapies and preventive strategies such as sulfadoxine–pyrimethamine chemoprevention in pregnant women. In this genomic surveillance study, dried blood spots and epidemiological data were collected from confirmed *Plasmodium falciparum* cases at 61 health facilities across all 10 Zambian provinces from March to July 2023. A total of 2,486 samples were genotyped by using multiplexed amplicon sequencing to identify mutations in 12 genes associated with resistance to seven antimalarial drugs. Several mutations potentially associated with artemisinin partial resistance were identified, including the validated kelch 13 (*k13*) P574L marker (0.66% adjusted national prevalence) and the candidate *k13* P441L marker (1.39%). The distribution of mutations was heterogeneous, with many health facilities reporting resistance markers in more than 5% of infections, and in some instances, up to 46% of infections. The multidrug resistance protein 1 N86 genotype, which is associated with decreased lumefantrine susceptibility, was found in all samples. Very high levels of sulfadoxine–pyrimethamine resistance markers were observed, including dihydropteroate synthetase K540E (93.26%). The variable prevalence of resistance markers underscores the need for routine molecular surveillance to detect emergent resistance and guide malaria control strategies. These results also call for studies designed to help us understand the clinical implications of these mutations and ensure the continued efficacy of antimalarial interventions in Zambia.

## INTRODUCTION

Between 2000 and 2015, malaria mortality and morbidity reduced globally, with cases declining from 79 to 58 per 1,000 population at risk.^1^ This progress was driven by the widespread adoption of effective vector control interventions (e.g., long-lasting insecticide-treated nets and indoor residual spraying), improved case management strategies (e.g., prompt diagnosis with rapid diagnostic tests [RDTs] and the effective treatment of malaria infections), and expanded access to preventative treatment for vulnerable populations (e.g., seasonal malaria chemoprevention and the intermittent treatment of pregnant women). Despite these gains, malaria remains a critical public health concern, particularly in sub-Saharan Africa, which accounts for ∼95% of global cases and deaths.^1^ Progress has plateaued because of a multitude of operational and biological challenges.^1^ Among these, the emergence of antimalarial drug resistance has been identified as a critical driver of malaria transmission, posing a significant threat to global malaria control and elimination efforts.^2^^,^^3^

Globally, antimalarial drug resistance has emerged against all frontline antimalarials, including artemisinin-based combination therapies (ACTs), which is the malaria treatment currently recommended by the WHO. Zambia, a country in southern Africa that exhibits highly heterogeneous rates of malaria transmission, was one of the first countries in sub-Saharan Africa to adopt the ACT, artemether–lumefantrine, as the first-line treatment for uncomplicated malaria in response to widespread resistance to chloroquine.^4^ Artemisinin partial resistance (ART-R), which is defined by delayed parasite clearance after treatment, is widespread in Southeast Asia and has recently emerged in East Africa, Central-East Africa, and the Horn of Africa.^5^^–^^8^ Resistance to ACT partner drugs appears to be limited in Africa, with recent data suggesting that artemether–lumefantrine and dihydroartemisinin–piperaquine, which have been used in mass treatment campaigns, remain efficacious in Zambia.^9^^,^^10^ Although sulfadoxine–pyrimethamine (SP)-resistant parasites are prevalent in the country, SP remains effective for the prevention of malaria during pregnancy in Zambia.^11^^–^^14^

Therapeutic and chemoprevention efficacy studies are crucial for monitoring drug effectiveness but are labor-intensive and costly. Molecular surveillance for mutations associated with antimalarial drug resistance can complement these studies by cost-efficiently quantifying spatiotemporal trends in drug resistance marker prevalence and identifying areas of concern that therapeutic efficacy studies could target. Certain mutations known as single-nucleotide polymorphisms (SNPs) in the *kelch* 13 (*k13*) gene result in amino acid changes in the propeller domain of the K13 protein that are associated with ART-R.^15^^,^^16^ Over the last decade, different *k13* SNPs associated with ART-R have emerged in the Horn of Africa, Central Africa, and East Africa.^7^^,^^8^^,^^17^^–^^20^ In contrast, chloroquine resistance is primarily mediated by a fixed set of mutations in the chloroquine resistance transporter (*crt*) gene and is modulated by mutations in the multidrug resistance protein 1 (*mdr1*) gene.^21^ Mutations in the *crt* and *mdr1* genes are also associated with resistance to piperaquine, amodiaquine, and lumefantrine, whereas copy number variations in the *mdr1* gene are linked to mefloquine resistance.^22^^,^^23^ An accumulation of specific SNPs in the dihydropteroate synthetase (*dhps*) and dihydrofolate reductase (*dhfr*) genes confer resistance to SP.^24^

Despite the high prevalence of *k13* SNPs and recent reports of delayed parasite clearance in the Horn of Africa, Central Africa, and East Africa, molecular surveillance and therapeutic efficacy studies in southern Africa are limited.^7^^,^^8^^,^^17^^–^^20^ In Zambia, up until 2018, molecular surveillance studies using either Sanger or molecular inversion probe deep sequencing rarely detected any *k13* mutations.^11^^,^^12^^,^^25^ Conversely, the *crt* K76T chloroquine resistance marker was found at low levels, whereas markers of resistance to SP were prevalent across the country.^11^^,^^12^^,^^26^^–^^28^ To provide a more comprehensive description of the current antimalarial drug resistance marker prevalence across southern Africa to help guide treatment policy and responses, the Genomics for Malaria in the Elimination 8 (GenE8) initiative implemented malaria molecular surveillance by using targeted amplicon deep sequencing in five southern African countries (Angola, Eswatini, Namibia, South Africa, and Zambia). In this study, we report on the prevalence of molecular markers associated with resistance to commonly used antimalarials in all 10 Zambian provinces based on falciparum-positive samples collected between March and July 2023.

## MATERIALS AND METHODS

### Study site.

Zambia is a landlocked country in southern Africa that neighbors the Democratic Republic of the Congo, Angola, Namibia, Botswana, Zimbabwe, Mozambique, Malawi, and Tanzania. Almost the entire population of 19 million (99%) is at risk of contracting malaria, with an average malaria prevalence of 29.3% among 2- to 10-year-olds.^9^ Malaria transmission is highly heterogenous, displaying a north-to-south gradient. The northern regions, including the Luapula (63.3%), North-Western (47.4%), Western (47.4%), and Muchinga Provinces (43.8%), exhibited the highest prevalence in 2021, whereas the Southern and Lusaka Provinces in the south are considered to be in the pre-elimination stage, with a prevalence of 3.3% (Figure 1A).^9^^,^^29^ Malaria transmission occurs year-round but peaks between January and April. Most (98%) infections are due to *Plasmodium falciparum* (*P. falciparum*) mono infections, with coinfections of *P. falciparum* and *Plasmodium malariae* (*P. malariae*), *Plasmodium ovale* (*P. ovale*), and *Plasmodium vivax* (*P. vivax*) occasionally reported.^9^^,^^30^^,^^31^ Common vectors include *Anopheles funestus*, *Anopheles gambiae* s.s., and *Anopheles arabiensis*.^9^ The healthcare system in Zambia includes 3,534 health facilities that serve as diagnostic and treatment centers for malaria. Malaria is diagnosed at point of care either via *P*. *falciparum* histidine-rich protein 2 (HRP2)-based RDTs or microscopy.^32^ All patients with uncomplicated malaria are treated with artemether–lumefantrine, whereas those with severe malaria are treated with intravenous artesunate.^9^^,^^32^ Dihydroartemisinin–piperaquine is reserved for mass drug administration in eliminating districts, whereas SP is used as an intermittent preventive treatment in pregnancy (IPTp).

**Figure 1.**
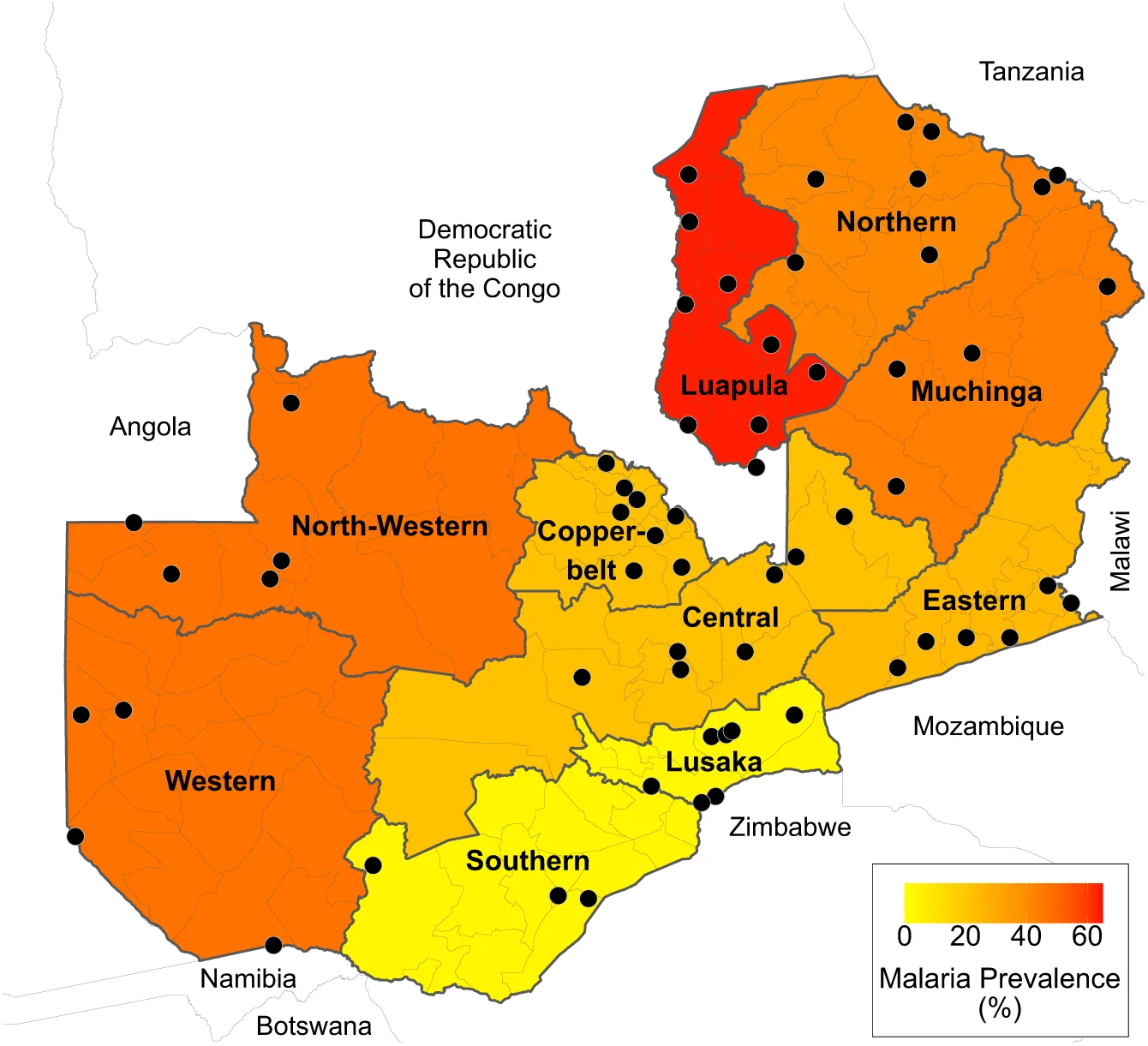
Study site. A map of Zambia with province-level malaria prevalence^29^ that indicates 61 health facilities with successfully sequenced samples (black dots).

### Study design, sampling, and data collection.

This cross-sectional health facility-based study was conducted as part of the wider GenE8 project, with sequenced samples collected as part of the Regional GenE8 project (ReGenE8) and contemporaneous samples obtained from other ongoing studies. The ReGenE8 project was nested within the national surveillance system and prioritized the representativeness of health facilities across geographies and predefined transmission strata. A minimum of 50 successfully sequenced samples from malaria RDT-positive patients were targeted at each of the 20 selected health facilities, which are distributed across all 10 provinces. To achieve this aim, 200 malaria RDT-positive patients were targeted for dry blood spot (DBS) and demographic data collection. The target sequencing sample size (*N* = 1,000) was intended to 1) estimate a 5% prevalence of a given molecular marker with a precision of 1.65% and 95% confidence and 2) achieve 90% power to detect a prevalence exceeding a value of 2% if the true prevalence is 3.9% by using a one-sided binomial test with ɑ = 0.05 and a design effect of 1.5. An additional 2,000 samples from 33 health facilities under a country-specific GenE8 project award and seven health facilities from a contemporaneous HRP2/3 surveillance project, were included to increase geographic representativeness. The achieved sample size of 2,013 sequenced infections across 61 health facilities provided a precision of 1.15% for a proportion of 5%, assuming a design effect of 1.5. The actual design effect was 14.60, and the intraclass correlation coefficient was 0.42.

Data and sample collection was conducted from March to July during the 2023 malaria transmission season in 67 participating health facilities (Figure 1A). All symptomatic patients over the age of 2 years who tested positive for falciparum malaria on an HRP2-based RDT (SD Bioline Malaria Ag P.f [Bioline, Memphis, TN] or First Response Malaria Antigen *P. falciparum* HRP2 Card tests [Premier Medical Corporation, Gujarat, India]) were invited to participate in the study. Patients with severe falciparum malaria, severe malnutrition, or febrile conditions attributable to nonmalarial diseases were excluded from the study.

Before enrollment in the study, written informed consent was obtained, as described in the Disclosures. Enrolled participants were interviewed at the point of care using CommCare (Dimagi, Cambridge, MA) or KoBo collect (KoBo Organization, Cambridge, MA) software on encrypted tablets to capture information on demographics, clinical symptoms, travel history, and occupation. Finger prick DBS samples collected on filter paper strips (Cytiva Whatman 3MM Chr [Cytiva, Marlborough, MA]) were labeled with unique barcodes to allow for the linking of genomic and survey data. The DBS samples were air-dried and stored individually in sealed bags with desiccant at –20°C until they were shipped to the regional sequencing hub at the National Institute for Communicable Diseases in South Africa or to the University of California, San Francisco.

### Sample processing and genomic assays.

DNA was extracted from at least 90 samples (one 6 mm punched DBS disk per sample) per health facility using the Chelex-Tween 20 method.^33^ Parasite densities were estimated using the *var* gene acidic terminal sequence (varATS) quantitative real-time polymerase chain reaction (qPCR) assay, with a minimum of 70 falciparum-positive samples from each health facility and a concentration of >100 parasites/µL randomly selected for downstream sequencing analysis.^34^ When this target sample size could not be achieved using the 100 parasites/µL cutoff, samples with parasitemias as low as 10 parasites/µL were included. Targeted amplicon sequencing libraries were prepared using MAD^4^HatTeR panel pools D1.1, R1.2, and R2.1, using CleanPlex reagents (Paragon Genomics, Fremont, CA), and sequenced using either a NextSeq 2000 (Illumina, San Diego, CA) or a NovaSeq X (Illumina) instrument using 150 paired-end reads.^35^ A custom bioinformatics pipeline was used to infer alleles across 241 targets.^35^ Sequences resulting from targets in the following genes are reported: *k13*, *crt*, *mdr1*, multidrug resistance protein 2 (*mdr2*), *dhps*, *dhfr*, *coronin*, apicoplast ribosomal protein S10 (*arps10*), phosphoinositide-binding protein 7 (*pib7*), ferrodoxin (*fd*), *exonuclease* (*exo*), and PF3D7_1322700. Microhaplotypes are reported for SNPs that are present within the same target. Variations to the original protocol and bioinformatic pipeline specifications are described in Eloff, Aranda-Díaz, et al.^36^

Further data cleaning, analysis, and visualization were conducted in R (version 4.3.1; R Foundation, Vienna, Austria). Samples with less than 10,000 reads, targets with <50 reads, and SNPs with a within-sample allele frequency (WSAF) <0.01 or ≤10 reads were excluded from the analysis. Subsequently, SNPs observed in only one sample at WSAF <0.01 and SNPs observed in multiple samples but exclusively in a single sequencing run were also excluded. Sequencing preparation batches with high contamination levels in either the negative or positive controls were excluded from all the analyses.

Non-falciparum targets were excluded from sequencing depth summaries. Otherwise, all resulting data were analyzed, including samples with partial coverage of SNPs, resulting in varying total sample numbers for each SNP assessed. Samples were considered successfully sequenced if a genotype was obtained for all 59 assessed *k13* SNPs. Multilocus haplotypes for polymorphisms across five independent targets in *dhfr*/*dhps* genes (*dhps* amino acids 436, 437, 540, 581, and 613; and *dhfr* amino acids 51, 59, 108 and 164), as well as three independent targets in the *mdr1* gene (amino acids 86, 184 and 1,246) were inferred for each sample by combining all individual haplotypes. Haplotypes were inferred for samples with either unmixed genotypes at all targets or with a single target showing a mixed genotype, in which case the sample was assumed to carry the two haplotypes accounting for the observed mixture. Haplotype classification for *dhps* (wild type, <2 mutations, double or triple), *dhfr* (wild type, <3 mutations, triple or quadruple), and *dhps*/*dhfr* (wild type, <5 mutations, quintuple or sextuple) are detailed in Supplemental Table 1. Samples with mixed genotypes in >2 amplicons were considered undetermined. A dataset that contains all individual genotypes is available online (DOI:10.5281/zenodo.15,119,253).

To evaluate patterns in the data and identify potential issues with sampling and library preparation, sample similarity was estimated using the root mean square error of WSAF for highly diverse targets (pool D1.1), followed by clustering by density-based spatial clustering of applications with noise, with ε = 0.2. Health facilities with possible DBS duplication were excluded from the analysis. These excluded facilities exhibited multiple clusters of samples with clonality >2 and nearly identical WSAF across all loci, suggesting that multiple DBSs were prepared using blood from a single patient.

## STATISTICAL ANALYSES

Descriptive statistics for sex, age group (<5, 5–14, 15–24, 25–39, 40–59, and >59 years old), occupation (including the self-identified category of “minor”), overnight travel in the past 2 months, and country of residence were calculated. To enable statistical analysis, some variables were reclassified: farming, herding, and fishing occupations were combined into a single occupational category (agricultural workers); individuals aged 25 years or older were grouped into a single category; and travel reports (within the 2 months prior to sample collection) were categorized as either international or domestic. The differences between successfully sequenced samples and qPCR-positive samples, as well as among provinces, were assessed using χ^2^ tests.

The proportion of samples carrying a mutation or haplotype (i.e., polyclonal samples with mixed genotypes counted as a carrier of the mutation) was calculated. Proportions of the *dhps*/*dhfr* haplotype were determined using the haplotype with the most mutations observed in each sample (Supplemental Table 1). The prevalence of infections carrying a genotype at the country and province levels were estimated, accounting for clustered sampling in provincial strata, and survey weights were applied to adjust for unequal sampling probabilities using the R “survey” package (v.4.4.2; https://cran.r-project.org/web/packages/survey/index.html). Additionally, 95% confidence intervals (CIs) were obtained by using the “beta” method.

Multivariate logistic regression was performed by using a generalized linear mixed-effects model to assess associations between the presence of markers of interest (as a binary outcome) and key categorical variables, including age group, sex, travel history, and occupation. A random intercept for each health facility and fixed effects for all other variables were included. This analysis was restricted to variables with sufficient sample size for convergence.

The complexity of infection (COI) for each sample and population allele frequencies were estimated jointly for each district and province by using moire v3.4.0.^37^ Microhaplotypes in all highly diverse *P*. *falciparum* targets (primer pool D1.1), as well as SNPs or microhaplotypes in drug resistance targets of interest, were used to infer COI and allele frequencies. The percentage of polyclonal infections was estimated as the mean of the individual probabilities of polyclonality.

Species-specific *lactate dehydrogenase* (*ldh*) targets in the panel were used to identify coinfections with non-*falciparum* species. The resulting sequences were used to remove unspecific amplification and to classify *P*. *ovale* reads into *P*. *ovale curtisi* or *P*. *ovale wallikeri*. Non-*falciparum* species were considered present if the combined reads from all *ldh* targets were >100, with each species contributing >1% of the total *ldh* targets reads.

## RESULTS

DNA was extracted from 4,737 samples, with sequencing libraries prepared for 3,084 (65.1%) samples. A total of 379 sequenced samples were excluded from further analysis: 274 samples from five health facilities suspected of DBS duplication, along with 105 samples from other health facilities, including all samples from one facility, because of potential cross-contamination. At least one SNP genotype was obtained for 91.9% (2,486/2,705) of the samples across 61 health facilities, with 2,013 (74.4%) successfully sequenced (with genotypes for all 59 *k13* SNPs).

Samples from all 10 provinces were successfully sequenced, although the sequencing success rates varied across provinces (Table 1). Luapula Province exhibited the highest number of successfully sequenced samples, whereas Western Province exhibited the lowest. The median per-sample sequencing depth was 719,499 reads (interquartile range [IQR]: 198,595–1,509,018), with a median per-target depth of 1,257 reads (IQR: 183–4,566). A median of 221 out of 238 targets (IQR: 186–225) had >100 reads. Single-nucleotide polymorphisms with the lowest genotyping success rates included *crt* 145, *crt* 218/220, and *mdr1* 86 (genotyped in 2,021, 1,015, and 2,023 samples, respectively). Nearly three-quarters of the samples analyzed (70.6%) were polyclonal, with a population mean COI of 3.05 (95% CI: 3.02–3.09; *N* = 2,446). Mixed genotypes in at least one drug resistance marker were detected in 52% of samples. Non-falciparum species, including *P*. *malariae*, *P*. *ovale curtisi*, *P*. *ovale wallikeri*, and *P*. *vivax*, were rare, being collectively observed in only 3.8% of samples (Supplemental Table 2).

**Table 1:** Demographics of participants with successfully sequenced samples

Characteristic	Overall	Western	North-Western	Southern	Copper-Belt	Lusaka	Central	Luapula	Northern	Muchinga	Eastern
*N*	2013	151	210	167	249	183	240	299	176	166	172
Health facilities	61	4	5	5	8	5	7	9	6	6	6
Age											
<5 years	217 (11%)	18 (12%)	15 (7.1%)	12 (7.2%)	32 (13%)	13 (7.1%)	14 (5.8%)	38 (13%)	24 (14%)	30 (18%)	21 (12%)
5–14 years	777 (39%)	64 (42%)	97 (46%)	83 (50%)	86 (35%)	81 (44%)	83 (35%)	106 (36%)	65 (37%)	64 (39%)	48 (28%)
15–24 years	574 (29%)	46 (30%)	65 (31%)	46 (28%)	69 (28%)	55 (30%)	79 (33%)	77 (26%)	50 (28%)	40 (24%)	47 (27%)
≥25 years	442 (22%)	23 (15%)	33 (16%)	26 (16%)	62 (25%)	34 (19%)	64 (27%)	77 (26%)	37 (21%)	30 (18%)	56 (33%)
Unknown	3	0	0	0	0	0	0	1	0	2	0
Sex											
Female	1,018 (51%)	76 (50%)	144 (69%)	81 (49%)	117 (47%)	84 (46%)	122 (51%)	153 (51%)	89 (51%)	73 (44%)	79 (46%)
Male	994 (49%)	75 (50%)	66 (31%)	86 (51%)	132 (53%)	99 (54%)	118 (49%)	145 (49%)	87 (49%)	93 (56%)	93 (54%)
Unknown	1	0	0	0	0	0	0	1	0	0	0
Occupation											
Agricultural	345 (19%)	17 (11%)	10 (7.6%)	24 (14%)	40 (16%)	32 (17%)	54 (23%)	79 (36%)	33 (19%)	25 (21%)	31 (18%)
Minor	776 (43%)	63 (42%)	81 (62%)	69 (41%)	115 (46%)	90 (49%)	79 (33%)	78 (36%)	85 (48%)	61 (51%)	55 (32%)
Other	147 (8.1%)	24 (16%)	7 (5.3%)	6 (3.6%)	33 (13%)	3 (1.6%)	10 (4.2%)	15 (6.9%)	14 (8.0%)	15 (13%)	20 (12%)
Student	241 (13%)	26 (17%)	8 (6.1%)	32 (19%)	34 (14%)	12 (6.6%)	35 (15%)	36 (17%)	20 (11%)	15 (13%)	23 (13%)
Unemployed	296 (16%)	21 (14%)	25 (19%)	36 (22%)	27 (11%)	46 (25%)	62 (26%)	9 (4.1%)	24 (14%)	3 (2.5%)	43 (25%)
Unknown	208	0	79	0	0	0	0	82	0	47	0
Country of residence											
Zambia	1,782 (99%)	151 (100%)	122 (93%)	167 (100%)	249 (100%)	183 (100%)	239 (100%)	215 (99%)	176 (100%)	119 (100%)	161 (94%)
Other	23 (1.3%)	0 (0%)	9 (6.9%)	0 (0%)	0 (0%)	0 (0%)	1 (0.4%)	2 (0.9%)	0 (0%)	0 (0%)	11 (6.4%)
Unknown	208	0	79	0	0	0	0	82	0	47	0
Reported travel (past 2 months)											
No travel	1,872 (96%)	148 (99%)	208 (100%)	158 (97%)	203 (94%)	179 (99%)	226 (97%)	251 (88%)	169 (97%)	165 (100%)	165 (96%)
Domestic	61 (3.1%)	1 (0.7%)	0 (0%)	5 (3.1%)	7 (3.3%)	1 (0.6%)	7 (3.0%)	32 (11%)	2 (1.1%)	0 (0%)	6 (3.5%)
International	1 (<0.1%)	0 (0%)	0 (0%)	0 (0%)	0 (0%)	0 (0%)	0 (0%)	0 (0%)	0 (0%)	0 (0%)	1 (0.6%)
Unspecified	10 (0.5%)	0 (0%)	0 (0%)	0 (0%)	5 (2.3%)	0 (0%)	0 (0%)	1 (0.4%)	4 (2.3%)	0 (0%)	0 (0%)
Unknown	69	2	2	4	34	3	7	15	1	1	0

A substantial proportion of the participants self-identified as minors (43%), whereas the most commonly reported occupations among adults were agricultural work (19%) and unemployment (16%). No significant differences in sex, travel history, or country of residence were observed between individuals with qPCR-positive samples and those with successfully sequenced samples (Table 1 and Supplemental Table 3). However, non-Zambian residents, participants over 25 years old, and participants from Southern and Eastern Provinces were underrepresented among the successfully sequenced samples. Most sequenced samples came from children and young adults (39% ages 5–14 and 29% ages 15–24) of either sex. Only a small proportion of participants were non-Zambian residents (1.3%) or reported any travel history (3.7%), with just one case of international travel recorded.

### Markers of artemisinin partial resistance.

Nonsynonymous mutations in 12 amino acids within the K13 propeller domain were detected in 225 samples (9.1% of all samples sequenced; Table 2). The candidate marker, P441L, the validated marker, P574L, and A578S, R622T, and P667A/S mutations were found in >0.5% of the sequenced samples. The validated marker, C469Y, was present in one sample from North-Western Province, whereas two samples, one in Western Province and one in Southern Province, carried the A675V marker. The R539I marker was found in five samples, including two in Western Province and three in North Western Province.

**Table 2:** Summary of observed non-synonymous mutations. Artemisinin partial resistance (ART-R) markers are categorized as validated or candidate following the current World Health Organization classification^38^

Gene	Observed in >0.5% of the Samples	Observed in ≤0.5% of the Samples
*k13*	Validated ART-R markers: P574L	Validated ART-R markers: C469Y, A675V
	Candidate ART-R markers: P441L	ART-R evidence lacking: K479I, S485N, N537S, R539I, Q613E
	ART-R evidence lacking: A578S, R622T, P667A, P667S	
*crt*		F48L, C72Y, M74I, N75E, K76T, A220S
*dhfr*	N51I, C59R, S108N, I164L	
*dhps*	S436A, A437G, K540E, A581G	S436F, A613S
*mdr1*	F184Y, D1246Y	N86Y

ART-R = artemisinin partial resistance; *crt* = chloroquine resistance transporter; *dhfr* = dihydrofolate reductase; *dhps* = dihydropteroate synthetase; *k13* = kelch 13; *mdr1* = multidrug resistance protein 1.

Variants in other genes can be found in Supplemental Table 8.

The candidate marker, P441L, was detected in 71 samples (3.1% of all samples sequenced) from 10 health facilities spanning nine districts and five provinces (Figure 2A and Supplemental Table 4), with an estimated national prevalence of 1.39% (CI: 0.45–3.25%; Figure 2B and Supplemental Tables 5 and 6). The mutation was most prevalent in the Central (5.32%; CI: 0.03–33.8%) and Western Provinces (3.69%; CI: 0–32.67%); however, the prevalence was less than 2.2% in the Southern, Lusaka, and North-Western Provinces. Students (odds ratio [OR]: 5.41; CI: 1.37–21.3, with respect to minors; Supplemental Table 7) and those who reported overnight domestic travel in the past 2 months were more likely to carry the P441L mutation (OR: 10.1; CI: 1.58–64.5). In the single health facility in the Chibombo District of Central Province, nearly half (47%; 46/98) of the analyzed samples carried the P441L mutation, with an allele frequency of 30.5% (CI: 23.7–38.3%; Figure 2A and Supplemental Figure 1).

**Figure 2.**
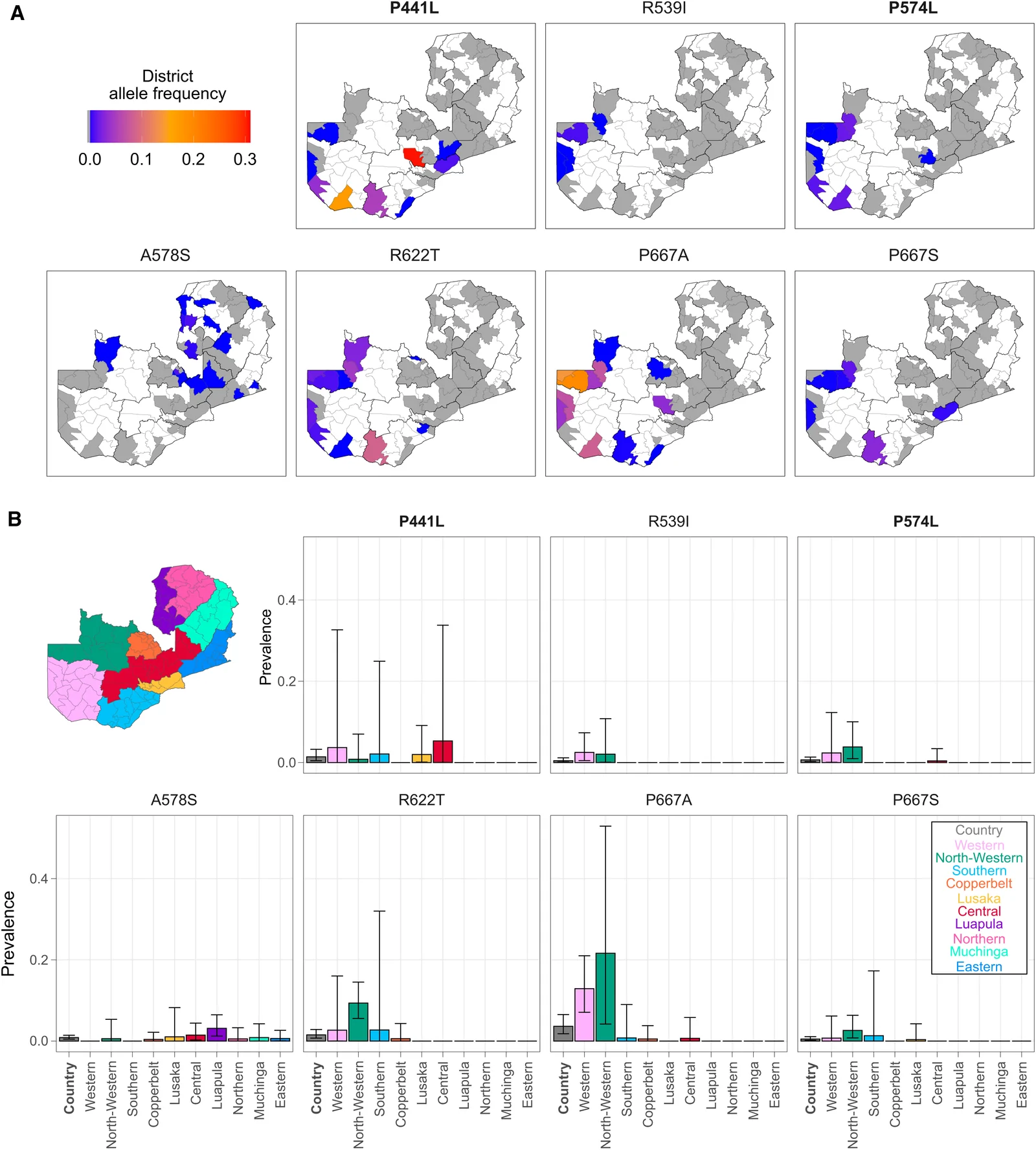
Allele frequency and prevalence of kelch 13 (*k13*) mutations. (**A**) District-level estimated allele frequencies for amino acids within *k13*. (**B**) National- and province-level estimated prevalence of infections carrying a mutation. Error bars indicate 95% CIs. Candidate (P441L) and validated (P574L) markers are indicated in bold. Only SNPs with variants in >0.5% of the samples are shown.

The P574L marker was found in 16 samples (0.6% of the total) across three provinces (Western, North-Western, and Central Provinces), with a national prevalence of 0.66% (CI: 0.27–1.34%). The North-Western and Western Provinces exhibited the highest proportions of P574L carriers and accounted for the majority of the observed R622T, R539I, P667A, and P667S mutations (Supplemental Tables 5 and 6). The P667A mutation reached a prevalence of 21.63% (CI: 4.2–52.91%) in North-Western Province, 12.91% (CI: 7.09–21.01%) in Western Province, and an estimated prevalence of 3.65% (CI: 1.80–6.53%) nationally. The 667A allele reached frequencies of 19.5% (CI: 10.6–31.6%) and 13.8% (CI: 8.5–20.9%) in the neighboring districts of Zambezi and Chavuma in North-Western Province. National prevalences of the R622T and P667S mutations were lower (1.54%; CI: 0.73–2.85%, and 0.51%; CI: 0.19–1.09%, respectively). The A578S mutation was predominantly found in the Eastern and Northern Provinces at a low national prevalence of 0.88% (CI: 0.50–1.43%). Allele frequencies mirrored prevalence patterns across these markers (Supplemental Table 4). There was no significant association between the carriage of any mutation (validated or candidate markers or mutations in the same amino acid as a validated or candidate marker) and demographic variables, including age, sex, or occupation (Supplemental Table 7).

Other polymorphisms previously described as a genetic background in the emergence of *k13* mutations in South East Asia, including *fd* D193Y, *arps10* V127M, and *mdr2* T484I, were not detected in any samples (Supplemental Table 8).^39^ Only two samples carried the *crt* I356T mutation, and one carried the *pib7* gene mutation C1484F (Supplemental Table 9). Similarly, *coronin* mutations G50E, R100K and E107V, which are associated with lower artemisinin resistance, were absent in the dataset.^40^

### Markers of SP resistance.

Mutations associated with SP resistance were prevalent throughout the country (Figure 3, Supplemental Figure 2, and Supplemental Tables 6 and 10–14). The SNPs within the triple *dhfr* mutant haplotype associated with pyrimethamine resistance, including *dhfr* N51I, C59R and S108N, all exhibited an estimated national prevalence greater than 90%. Southern Province exhibited the lowest *dhfr* N51I (81.59%; CI: 64.42–92.82%) and *dhfr* C59R (76.97%; CI: 50.79–93.41%) SNP prevalence. The *dhfr* S108N SNP was present in all samples, although three (0.86%) samples from Luapula Province exhibited a mixed genotype. Only two samples in Muchinga Province carried *dhfr* I164L, a mutation that, when found in a quadruple haplotype with the triple mutants, confers higher pyrimethamine resistance. Of the 2,379 samples with genotypes available in all four *dhfr* SNPs, 0.13% were undetermined (Supplemental Figure 2 and Supplemental Table 12). Of the 2,376 unambiguous samples, the triple mutant was found in 96.89%, and the quadruple mutant was found in 0.08% (Figure 3C).

**Figure 3.**
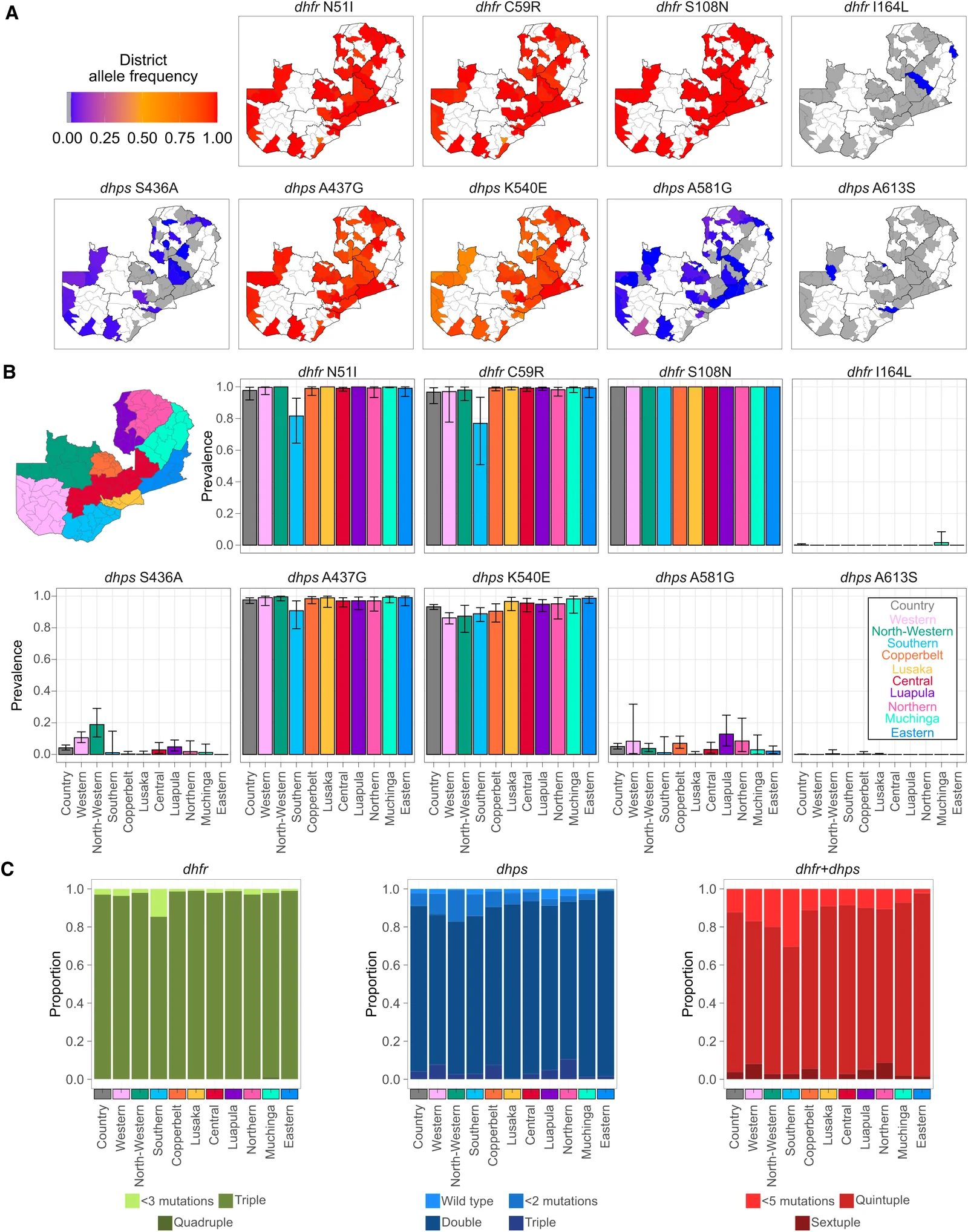
Allele frequency and prevalence of dihydrofolate reductase (*dhfr*) and dihydropteroate synthetase (*dhps*) mutations. (**A**) District-level estimated allele frequencies for amino acids within *dhfr* and *dhps*. (**B**) National- and province-level estimated prevalence of infections carrying a mutation. Error bars indicate 95% CIs. Only SNPs with variants in >0.5% of the samples are shown. (**C**) Proportion of samples carrying *dhfr*, *dhps*, or *dhfr*/*dhps* haplotypes. Each sample was assigned the haplotype with the most mutations in the sample (Supplemental Table 1). Polyclonal samples with mixed genotypes in multiple targets were undetermined and excluded.

Of the *dhps* double mutant haplotype SNPs associated with sulfadoxine resistance, including *dhps* S436A/F or A437G and K540E, the *dhps* A437G and *dhps* K540E mutations exhibited a national prevalence of 97.62% (CI: 95.37–98.96%) and 93.26% (CI: 91.59–94.69%), respectively. The *dhps* S436A mutant was found at a 4.17% prevalence (CI: 2.80–5.96%) nationally, whereas S436F was very rare (0.06%; CI: 0–0.28%). The prevalence of the *dhps* A437G mutation was lowest in Southern Province (90.78%; CI: 79.42–97.04%), whereas *dhps* S436A was most prevalent in the North-Western (18.87%; CI: 11.02–29.12%) and Western Provinces (10.52%; CI: 7.48–14.27%). The *dhps* S436F mutation was only detected in the Copperbelt and Lusaka Provinces. The *dhps* K540E mutation exhibited the lowest prevalence (<90%) in the Western, North-Western, and Southern Provinces. Two additional *dhps* mutations (A581G or A613S) associated with higher sulfadoxine resistance when part of a triple mutant haplotype were found. The A581G mutation was found at 4.99% (CI: 3.44–6.98%) nationally, with the highest prevalence in Luapula Province (12.87%; CI: 5.55–24.2%) and a single health facility in the Shesheke District, Western Province, where it reached a proportion of 41.38% (12/29) and an allele frequency of 22.1% (CI: 11.8–35.6%). The A613S mutation was relatively rare, found in the North-Western, Lusaka, and Copperbelt Provinces at prevalences below 0.5%. Of the 2,257 samples with genotypes available in all five SNPs, 13.51% were undetermined (Supplemental Figure 2 and Supplemental Table 13). Of the 1,952 unambiguous samples, the double mutant haplotype was found in 87.04%, and the triple mutant was found in 4.15% (Figure 3C). Reconstructing the full *dhps*/*dhfr* haplotype for 1,869 unambiguous samples revealed the quintuple mutant haplotype (*dhfr* triple and *dhps* double mutant haplotypes) in 84.02% and the sextuple mutant haplotype (*dhfr* quadruple and *dhps* double or *dhfr* triple and *dhps* triple mutant haplotypes) in 3.90% (Figure 3C, Supplemental Figure 2, and Supplemental Table 14).

### Markers of resistance to other antimalarial drugs.

Haplotypes in *mdr1* 86/184/1246 were successfully inferred for 2,011 of 2,022 samples (99.46%), with valid genotypes at all three codons (Supplemental Table 15). The *mdr1* N86/Y184/D1246 (NYD) haplotype was the most common, detected as a pure allele in 46.94% of samples and mixed with *mdr1* N86/Y184F/D1246 (NFD) haplotype in 34.44%. The pure NFD haplotype was observed in 18.10% of samples. The *mdr1* N86 “wild type” genotype, which is linked to lower lumefantrine susceptibility, was found in all samples.^23^ The *mdr1* N86Y mutation, which is associated with resistance to amodiaquine and chloroquine, was detected in only four samples, for which a haplotype could not be inferred. The *mdr1* Y184F mutation exhibited a prevalence of 50.08% (CI: 42.63–57.53%; Supplemental Table 6), whereas the *mdr2* I492V mutation exhibited a prevalence of 49.43% (CI: 45.49–53.37%; Supplemental Table 6). District-level allele frequencies ranged from 37.5% to 98.1% for *mdr1* Y184F and from 8.7% to 57.3% for *mdr2* I492V (Supplemental Table 16). Lastly, the chloroquine-resistant *crt* C72/V73/M74I/N75E/K76T (CVIET) microhaplotype exhibited a low national prevalence of 0.18% (CI: 0.04–0.50%; Supplemental Table 9).

## DISCUSSION

In this study, we investigated the prevalence of antimalarial drug resistance markers across all 10 provinces of Zambia by using multiplexed amplicon deep sequencing. Mutations in the *k13* gene, which may impact artemisinin efficacy, were identified in six provinces. Validated markers, such as C469Y, P574L, and A675V, along with the candidate marker, P441L, were detected at low national prevalences (<3%) but showed notable geographic variation, with higher frequencies in specific health facilities and districts. Additionally, other *k13* mutations, including R622T and P667A/S, were observed. The *mdr1* N86 wild-type genotype, which is associated with reduced lumefantrine susceptibility, was present in all samples, whereas markers for SP resistance were widespread.^23^ Low-level coinfections with *P*. *malariae*, *P*. *ovale curtisi*, *P*. *ovale wallikeri*, and *P*. *vivax* were also detected.

Concerns around the emergence of partial artemisinin resistance in Africa have been driven by the rapid emergence of a diverse array of *k*13 mutations, including A675V, C469Y, P574L, and P441L, in the past decade in East Africa and the Horn of Africa.^8^^,^^17^^–^^20^^,^^41^^,^^42^ The *k13* A675V and C469Y markers, found in two and one Zambian samples, respectively, have increased in prevalence in Uganda since 2016.^18^ In contrast, the validated marker, P574L, which is rarely reported on the continent, was observed in 16 samples across three provinces in this study^43^^,^^44^ The candidate marker, P441L, which was identified in 71 samples, has previously been reported in a few samples in Zambia’s North-Western Province and the Democratic Republic of the Congo and has recently increased in western Uganda.^18^^,^^25^^,^^45^ At the time of writing, a new study has reported the emergence of P441L in Zambia’s Choma District, Southern Province, with its prevalence rising from none in 2018 to 7.2% in 2023, which is consistent with our findings.^46^ A high proportion of P441L carriers has also been reported within the GenE8 project in Namibia’s Zambezi Region, which borders the Southern and Western Provinces.^36^ Notably, the clinical and phenotypical effects of the P574L and P441L mutations on African parasites have not yet been evaluated.^2^ The P441L mutation, in particular, lacks robust clinical and laboratory evidence linking it to delayed clearance; however, a recent study in Myanmar observed an increase after mass drug administration with dihydroartemisinin–piperaquine.^47^ Other candidate or validated markers of ART-R that have been observed on the continent, such as R561H, C469F, and R622I, were not found in this study.^8^^,^^17^^,^^19^^,^^20^^,^^42^ However, we did find a high prevalence of R622T and P667A mutations, which occur in the same amino acids as the validated marker, R622I, and the mutations, P667T/S, which exhibit limited evidence of delayed clearance.^8^^,^^48^^,^^49^ The A578S mutation, which is widespread in Africa but does not impact artemisinin susceptibility, was observed at low levels and generally in areas in which other *k13* mutations were not observed.^50^ This separation might reflect parasite population structure or differing genomic backgrounds within the country. Further studies on parasite relatedness and the A578S phenotype are needed to elucidate this pattern. The effect of other *k*13 mutations identified in this study that are potentially associated with delayed clearance also remains understudied.^25^

Clear geographic trends in *k*13 mutations were shown in this study, with all mutations of interest observed in provinces in the western half of the country. The highest proportion of the P441L mutation was observed in the Chibombo District, Central Province, which is an intermediate transmission setting. Artemisinin partial resistance has generally emerged in low transmission settings or areas experiencing a surge in transmission, where the exposure of nonimmune individuals can drive drug pressure because of treatment-seeking behavior and decrease the likelihood of clearing infections with resistant parasites.^18^ Furthermore, areas with low transmission are typically associated with lower intra-host genetic diversity, which would reduce within-host parasite competition and increase the likelihood of resistant strains persisting.^51^^,^^52^ The P441L and P574L markers, as well as R622T and P667A/S, were identified in this study in intermediate transmission settings in Zambia, including a health facility near a Namibian facility where similar results were recently reported.^36^ These settings also exhibited higher levels of mixed polyclonal infections, which is consistent with the expected higher parasite genetic diversity in areas of intermediate malaria transmission. The interplay of factors, including host immunity, pharmacokinetics, parasite physiology, epidemiology, and treatment-related non-intrinsic factors, in driving the selection and spread of these mutations remains to be fully elucidated.

The origin of these mutations remains unclear. They may have emerged de novo in Zambia or Namibia and spread locally or been introduced from regions such as Eastern Africa, where these mutations are becoming more common.^2^^,^^36^^,^^46^ Human mobility, both within Zambia and across its borders, likely plays a critical role in the introduction and spread of resistant parasites. Currently, limited data on cross-border movement in Zambia’s northeast makes it challenging to quantify the risk of introducing parasites carrying mutations from East Africa. The higher transmission intensity in the northeast may also limit onward spread, even if introductions occur. Additional data on human mobility and parasite whole-genome sequences would improve our understanding of the origins and pathways of these resistant strains within the country.

The presence of ART-R does not necessarily result in ACT failure, as the partner drug can clear remaining parasites from the bloodstream. However, ART-R can compromise the efficacy of monotherapies used in severe malaria treatment and increase selection pressure for resistance to partner drugs. Currently, there is no strong evidence of full resistance to lumefantrine or pyronaridine, although resistance to other partner drugs, such as amodiaquine, mefloquine, and piperaquine, has been documented in South East Asia.^2^ In contrast, parasites in Africa generally lack the genotypes associated with resistance and have demonstrated continued high susceptibility in ex vivo studies.^53^^,^^54^ Decreased susceptibility to lumefantrine is associated with certain polymorphisms, including *crt* K76 and *mrd1* N86, both of which were observed in nearly all samples in this study.^23^^,^^53^ This likely reflects Zambia’s widespread use of artemether–lumefantrine for the treatment of uncomplicated malaria. The *mdr1* 86/184/1246 NFD haplotype, which was previously linked to reduced lumefantrine sensitivity and selection via artemether–lumefantrine treatment, was predominant alongside NYD, with the *mdr1* Y184F mutation showing a prevalence of 50.08%.^55^^–^^57^ Given Zambia’s widespread use of artemether–lumefantrine, the high prevalence of *mdr1* N86 observed likely reflects drug pressure; however, the absence of the dominance of the NFD haplotype suggests that further investigation into the role of *mdr1* Y184F in lumefantrine susceptibility is required. Although modest changes in lumefantrine susceptibility have been reported in East Africa, they are unlikely to affect the efficacy of ACTs.^53^^,^^54^ No SNPs associated with resistance to amodiaquine or piperaquine were detected in this study. However, we did not assess copy number variations in *mdr1* and *plasmepsin* II/III, which confer resistance to mefloquine and piperaquine.^58^^,^^59^

Mutations associated with SP resistance were highly prevalent in this study. Although the full *dhps*/*dhfr* haplotype could not be determined in all samples because of sequencing depth variability, the quintuple mutant haplotype, which is a strong predictor of SP treatment failure, was detected in a majority of the samples. Its hallmark SNP, *dhps* K540E, was found at a prevalence exceeding 90%.^60^ Although SP is not used as a partner drug for malaria treatment in Zambia, it is widely administered as an IPTp, with ∼90% of pregnant women receiving at least one dose.^29^ Notably, Southern Province, which reported the lowest IPTp coverage (68.4%), also exhibited the lowest prevalence of *dhfr* triple mutant haplotypes, largely due to a very low proportion of mutants in Pemba District.^29^ These findings suggest that SP is unlikely to be efficacious for malaria treatment nationwide and should not be considered as a partner drug in ACT regimens. Despite the high prevalence of molecular resistance, SP remains effective for IPTp in Zambia because multiple doses improve birth outcomes and clear infections, even in settings with a high prevalence of the quintuple mutant haplotype.^13^^,^^14^^,^^61^ Although the effects of sextuple mutant haplotypes SNPs, including *dhps* A581G, *dhps* A613S, and *dhfr* I164L, on SP’s chemopreventive efficacy remain poorly understood, a recent study has revealed that the presence of the *dhps* A581G mutation is associated with reduced birth weights.^62^ These SNPs were observed at an overall prevalence of ∼5% but reached higher levels in specific districts within the Luapula and Western Provinces. Given SP’s importance in improving maternal health outcomes and its role in reducing selective pressure on ACT components, the efficacy of SP for IPTp should be closely monitored.

Parasite migration has played a critical role in the emergence and spread of antimalarial drug resistance in Africa and globally. For instance, chloroquine resistance first appeared in Southeast Asia and South America before rapidly spreading worldwide.^63^ Similarly, SP-resistant haplotypes originated in Southeast Asia and later migrated to Africa.^64^ In contrast, some mutations associated with ART-R have arisen independently in both Southeast Asia and Africa.^7^^,^^65^ The dynamics and drivers of ART-R spread in Africa remain poorly understood. Specific ART-R molecular markers have been observed across borders in East Africa and the Horn of Africa, with sporadic reports elsewhere.^8^^,^^16^^–^^20^^,^^41^^,^^42^^,^^66^ Provinces in Zambia reporting *k13* mutations border Angola, Botswana, Mozambique, Namibia, and Zimbabwe, highlighting the potential role of parasite migration. Non-Zambian residents were captured in this study only in the North-Western and Eastern Provinces, but their numbers were insufficient for robust statistical analysis. The observed association between the *k13* P441L mutation and reported travel, which was almost exclusively domestic, is based on a limited number of cases and should be interpreted with caution. Previous research from Namibia’s Zambezi Region, which borders Zambia’s Southern and Western Provinces, underscored the role of human migration and parasite migration on malaria epidemiology in Namibia.^67^^,^^68^ Our findings on the spread of *k13* mutations in southwestern Zambia and northern Namibia align with these findings.^36^ Understanding the role of domestic and international human mobility in malaria transmission in Zambia is under-researched but critical for developing targeted strategies to limit the spread of resistance genotypes. On a larger scale, the relatedness of parasites that carry *k13* mutations in East Africa, the Horn of Africa, and Southeast Asia to those found in this study remains unknown.

This study had several limitations. Samples were collected over part of a single season, limiting our ability to identify temporal trends. The sampling was designed to estimate country-level prevalence with broad geographical representation but was not powered to detect fine-scale geographical patterns. Although most health facilities were randomly selected within provinces, some sentinel sites were intentionally added, potentially violating statistical assumptions underlying survey weights to adjust for sampling bias. In general, province estimates are likely to be the most reliable. In addition, low levels of most mutations and incomplete data for some demographic variables reduced the sample size available for evaluating associations between mutations and demographics. Allele frequencies were only estimated at the district level given that the panmixia assumption is unlikely to hold at larger scales. Technical limitations of the molecular assay included challenges in phasing SNPs from independent amplicons for haplotype construction, which could be mitigated by using long amplicon sequencing approaches.

Molecular approaches are increasingly recognized as essential surveillance tools for guiding programmatic decision-making in malaria control and elimination. By using multiplex amplicon sequencing and a geographically comprehensive sampling approach, concerning levels of *k*13 mutations associated with ART-R across multiple Zambian provinces and transmission strata were identified in this study. Furthermore, these data were generated in a timely manner from samples collected in 2023, the results of which were shared with the National Malaria Elimination Centre in near real-time starting in early 2024 and published in early 2025. Although the impact of the mutations identified in this study on resistance and treatment efficacy is unclear, their high frequencies in certain health facilities and districts, coupled with consistent increases in the P441L mutation noted in other studies, suggest they may confer a selective advantage in a country that predominantly uses ACTs for treatment and interventions. Targeted therapeutic efficacy and ex vivo susceptibility studies, as well as longitudinal and geographically representative molecular surveillance, will be necessary to fully understand the implications of these mutations and monitor their spread. Decision-makers must also prioritize strategies to prevent the emergence and limit the spread of partner drug resistance and other ART-R genotypes, thereby safeguarding treatment efficacy and progress toward elimination goals.

## Supplemental Materials

10.4269/ajtmh.25-0110 Supplemental Materials
