# Supplementary material for: Plasmodium falciparum Genomic Surveillance Reveals a Diversity of Kelch 13 Mutations in Zambia
Source: Am J Trop Med Hyg. 2025 Jul 31;115(5 Suppl):65–76. doi: 10.4269/ajtmh.25-0110 (PMC13736986; doi:10.4269/ajtmh.25-0110)
Supplement: Supplementary file 1 [file tpmd250110.SD1.pdf]

Supplemental Materials

Plasmodium falciparum genomic surveillance reveals a diversity of *kelch13* mutations in Zambia

List of supplementary figures and tables:

Figure S1. Proportion of samples carrying a *k13* mutation in each of the 61 health facilities.
Figure S2. Proportion of samples with *dhfr* and *dhps* haplotypes including undetermined samples.
Table S1: *Dhps*/*dhfr* haplotype classification.
Table S2. Proportion of *P*. *falciparum* infections and coinfections with non-*falciparum* species.
Table S3. Demographic characteristics of participants whose samples were qPCR-positive and successfully sequenced.
Table S4: District-level *k13* mutation allele frequencies.
Table S5: Proportion of samples carrying non-synonymous and synonymous *k13* mutations.
Table S6: Adjusted prevalence for mutations observed in >0.5% of the samples.
Table S7: Multivariate logistic regression of select demographic characteristics and genotypes.
Table S8: Non-synonymous mutations not reported in Table 2.
Table S9: Proportion of samples carrying a non-synonymous or synonymous mutation in *crt*, *exo*, *mdr2*, PF3D7_1322700 and *pib7*.
Table S10: District-level *dhfr* microhaplotype allele frequencies.
Table S11: District-level *dhps* microhaplotype allele frequencies.
Table S12: Proportion of samples carrying a *dhfr* haplotype.
Table S13: Proportion of samples carrying a *dhps* haplotype.
Table S14: Proportion of samples carrying a *dhps-dhfr* haplotype.
Table S15: Proportion of samples carrying an *mdr1* haplotype.
Table S16: District-level *mdr1* and *mdr2* allele frequencies.

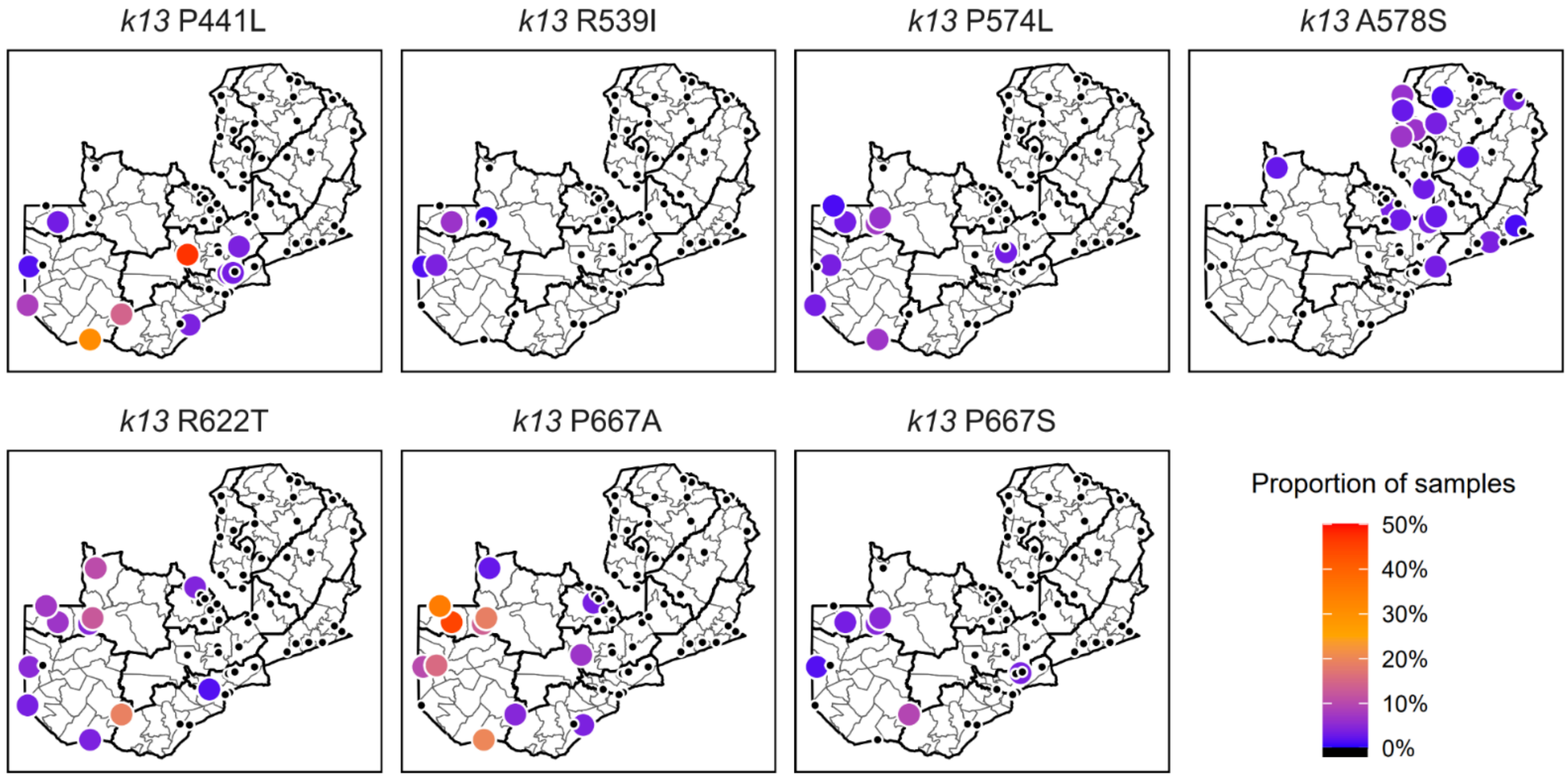

Figure S1. Proportion of samples carrying a *k13* mutation in each of the 61 health facilities. Health facilities with non-zero proportions are shown in larger colored dots.

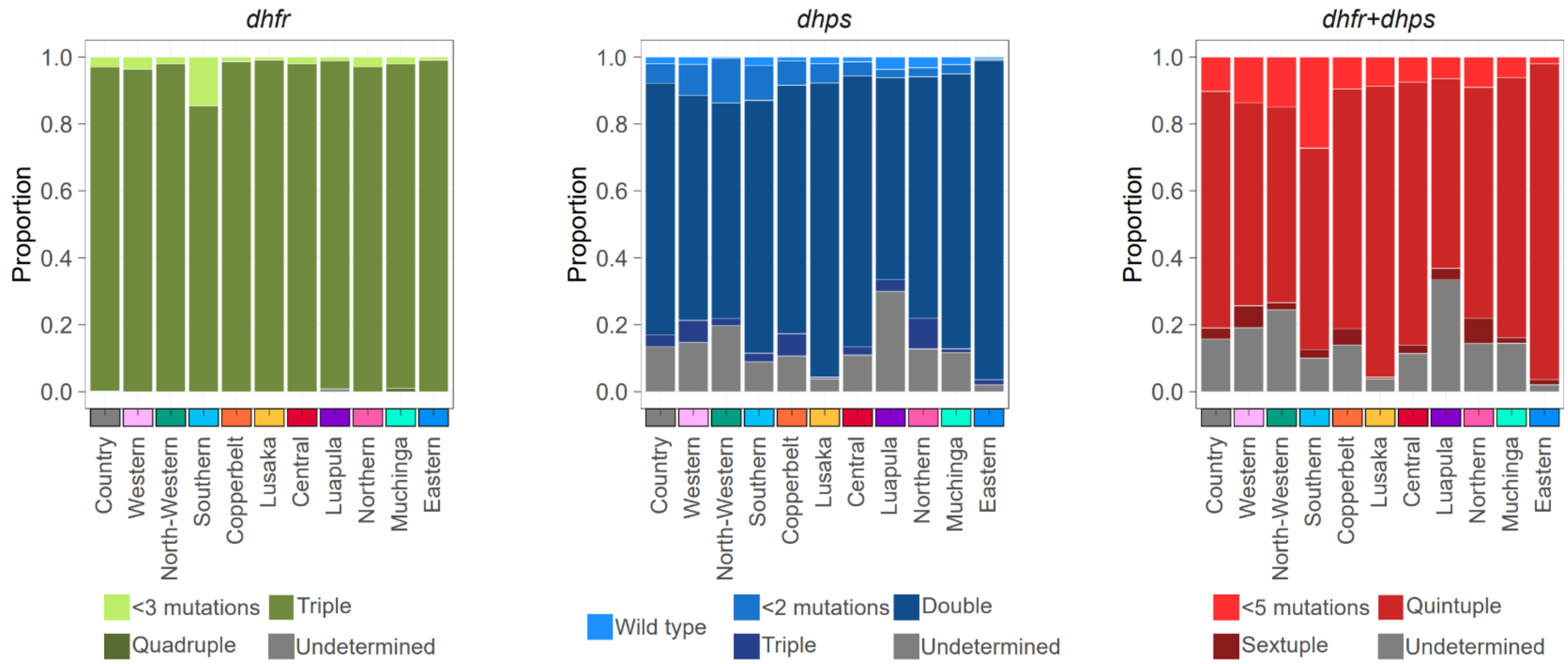

Figure S2. Proportion of samples with *dhfr* and *dhps* haplotypes including undetermined samples. Proportion of samples carrying *dhfr*, *dhps*, or *dhfr*/*dhps* haplotypes. Each sample was assigned the haplotype corresponding to the highest level of resistance detected. Same as Figure 3C but including polyclonal samples with mixed genotypes in multiple targets classified as undetermined.

Table S1: *Dhps*/*dhfr* haplotype classification.

Gene (amino acids)	Haplotype classification	Pure genotypes	Observed mixed genotypes under classification
dhps<br>(431,436,437,540,581,613)	Wild type	ISAKAA	
	<2 mutations	IAAKAA<br>IAGKAA<br>ISAEAA<br>ISGKAA	IAAKAA+ISGKAA<br>IAGKAA+ISGKAA<br>ISAEAA+ISAKAA<br>ISAKAA+ISGKAA
	Double	ISGEAA	IAGEAA+ISAEAA+ISGEAA<br>IAGEAA+ISGEAA<br>ISAEAA+ISGEAA<br>ISGEAA+ISGEAV<br>ISGEAA+ISGKAA
	Triple	ISGEGA	ISGEAA+ISGEAS<br>ISGEAA+ISGEGA<br>ISGEAA+ISGEGA+ISGKAA<br>ISGEGA+ISGKAA
dhfr<br>(16,51,59,108,164)	Wild type	ANCSI	
	<3 mutations	AICNI<br>ANRNI<br>ANCNI	AICNI+ANRNI
	Triple	AIRNI	AICNI+AIRNI<br>AICNI+AIRNI+ANRNI<br>AIRNI+ANRNI<br>AIRNI+AIRSI
	Quadruple	AIRNL	AIRNI+AIRNL
dhps-dhfr	Wild type	ISAKAA-ANCSI	
	<5 mutations	IAGKAA-AIRNI<br>ISAKAA-AIRNI<br>ISGEAA-AICNI<br>ISGEAA-ANRNI<br>ISGKAA-AICNI<br>ISGKAA-AIRNI<br>IAAKAA-AIRNI<br>ISAKAA-AICNI<br>ISAEAA-AIRNI<br>ISGEAA-ANCNI<br>ISGEGA-AICNI<br>ISGKAA-ANRNI<br>ISAKAA-ANRNI	IAGKAA-AIRNI+ISGKAA-AIRNI<br>ISAKAA-AIRNI+ISGKAA-AIRNI<br>ISAKAA-AICNI+ISGKAA-AICNI<br>ISGKAA-AICNI+ISGKAA-AIRNI<br>IAGKAA-AICNI+IAGKAA-AIRNI<br>IAAKAA-AIRNI+ISGKAA-AIRNI<br>ISGEAA-AICNI+ISGEGA-AICNI<br>ISGEAA-AICNI+ISGEAA-ANRNI<br>ISAEAA-AIRNI+ISAKAA-AIRNI<br>ISGEAA-AICNI+ISGKAA-AICNI
	Quintuple	ISGEAA-AIRNI	ISAEAA-AIRNI+ISGEAA-AIRNI<br>ISGEAA-AICNI+ISGEAA-AIRNI<br>ISGEAA-AIRNI+ISGEAA-ANRNI<br>ISGEAA-AIRNI+ISGKAA-AIRNI<br>ISGEAA-AIRNI+ISGEAA-AIRSI<br>ISGEAA-AICNI+ISGEAA-AIRNI+ISGEAA-ANRNI<br>ISGEAA-AIRNI+ISGEAV-AIRNI<br>IAGEAA-AIRNI+ISGEAA-AIRNI
	Sextuple	ISGEGA-AIRNI<br>ISGEAS-AIRNI<br>ISGEAA-AIRNL	ISGEAA-AIRNI+ISGEGA-AIRNI<br>ISGEAA-AIRNI+ISGEGA-AIRNI+ISGKAA-AIRNI<br>ISGEAA-AIRNI+ISGEAS-AIRNI<br>ISGEAA-AIRNI+ISGEAA-AIRNL<br>ISGEGA-AIRNI+ISGKAA-AIRNI

Table S2. Proportion of *P. falciparum* infections and coinfections with non-*falciparum* species

	Overall (*N* = 2486)	Central (*N* = 314)	Copperbelt (*N* = 297)	Eastern (*N* = 207)	Luapula (*N* = 354)	Lusaka (*N* = 214)	Muchinga (*N* = 197)	North Western (*N* = 252)	Northern (*N* = 206)	Southern (*N* = 241)	Western (*N* = 204)
**Pf**	2391 (96.18%)	307 (97.77%)	287 (96.63%)	206 (99.52%)	323 (91.24%)	213 (99.53%)	189 (95.94%)	229 (90.87%)	200 (97.09%)	240 (99.59%)	197 (96.57%)
**Pf+Pm**	41 (1.65%)	0 (0%)	4 (1.35%)	0 (0%)	15 (4.24%)	0 (0%)	3 (1.52%)	15 (5.95%)	2 (0.97%)	1 (0.41%)	1 (0.49%)
**Pf+Pm+ Poc**	5 (0.2%)	0 (0%)	0 (0%)	0 (0%)	3 (0.85%)	0 (0%)	0 (0%)	0 (0%)	1 (0.49%)	0 (0%)	1 (0.49%)
**Pf+Pm+ Poc+Pow**	1 (0.04%)	0 (0%)	0 (0%)	0 (0%)	0 (0%)	0 (0%)	0 (0%)	0 (0%)	0 (0%)	0 (0%)	1 (0.49%)
**Pf+Poc**	24 (0.97%)	3 (0.96%)	5 (1.68%)	1 (0.48%)	6 (1.69%)	0 (0%)	4 (2.03%)	3 (1.19%)	2 (0.97%)	0 (0%)	0 (0%)
**Pf+Poc+ Pow**	2 (0.08%)	0 (0%)	0 (0%)	0 (0%)	1 (0.28%)	0 (0%)	0 (0%)	1 (0.4%)	0 (0%)	0 (0%)	0 (0%)
**Pf+Pow**	19 (0.76%)	2 (0.64%)	1 (0.34%)	0 (0%)	6 (1.69%)	1 (0.47%)	1 (0.51%)	4 (1.59%)	1 (0.49%)	0 (0%)	3 (1.47%)
**Pf+Pv**	3 (0.12%)	2 (0.64%)	0 (0%)	0 (0%)	0 (0%)	0 (0%)	0 (0%)	0 (0%)	0 (0%)	0 (0%)	1 (0.49%)

Pf: *P. falciparum*; Pm: *P. malariae*; Poc: *P. ovale curtisi*; Pow: *P. ovale wallikeri*; Pv: *P. vivax*

Table S3. Demographic characteristics of participants whose samples were qPCR-positive and successfully sequenced

Characteristic	qPCR-positive	Successfully sequenced	*P*-value*
**Age**	*N* = 4220	*N* = 2010	**0.01**
< 5 years	10.1%	10.8%	
5 - 14 years	35.9%	38.7%	
15 - 24 years	28.2%	28.6%	
≥25 years	25.8%	22%	
**Sex**	*N* = 4223	*N* = 2012	0.86
Female	50.9%	50.6%	
Male	49.1%	49.4%	
**Occupation**	*N* = 3864	*N* = 1805	0.07
Agricultural	22%	19.1%	
Minor	40.7%	43%	
Other	8.9%	8.1%	
Student	12.1%	13.4%	
Unemployed	16.3%	16.4%	
**Country of residence**	*N* = 3866	*N* = 1805	**<0.001**
Zambia	96.4%	98.7%	
Other	3.6%	1.3%	
**Reported travel**	*N* = 4114	*N* = 1944	**0.5**
No Travel	96.7%	96.3%	
Domestic	2.5%	3.1%	
International	0.3%	0.1%	
Unspecified	0.6%	0.5%	
**Province**	*N* = 4225	*N* = 2013	**0.02**
Central	11.3%	11.9%	
Copperbelt	11.6%	12.4%	
Eastern	10.7%	8.5%	
Luapula	13.7%	14.9%	
Lusaka	7.5%	9.1%	
Muchinga	8.4%	8.2%	
North Western	10.2%	10.4%	
Northern	9.1%	8.7%	
Southern	10.5%	8.3%	
Western	7.1%	7.5%	

* χ^2 test

Table S4: District-level *k13* mutation allele frequencies. Source data for Figure 2A.

Province	District	HFs[a]	Samples[b]	*k13* 441		*k13* 539		*k13* 574		*k13* 578		*k13* 622		*k13* 667		
				L	P	I	R	L	P	A	S	R	T	A	P	S
Western	Kalabo	1	27	0	1	3.363e-08 (8.472e-09 - 5.613e-08)	1 (1 - 1)	4.266e-08 (3.723e-08 - 5.342e-08)	1 (1 - 1)	1	0	1	0	0.066 (0.017 - 0.167)	0.934 (0.833 - 0.983)	0
Western	Sesheke	1	29	0.168 (0.081 - 0.293)	0.832 (0.707 - 0.919)	0	1	0.012 (3.866e-08 - 0.068)	0.988 (0.932 - 1)	1	0	1 (1 - 1)	3.437e-09 (0e0 - 3.437e-09)	0.078 (0.023 - 0.179)	0.922 (0.821 - 0.977)	0
Western	Shan-gombo	1	87	0.035 (0.012 - 0.076)	0.965 (0.924 - 0.988)	0	1	0.011 (0.001 - 0.034)	0.989 (0.966 - 0.999)	1	0	0.994 (0.968 - 1)	0.006 (0 - 0.032)	0	1	0
Western	Sikongo	1	61	4.986e-08 (3.330e-08 - 5.816e-08)	1 (1 - 1)	2.060e-09 (0e0 - 4.533e-08)	1 (1 - 1)	0	1	1	0	0.987 (0.959 - 0.998)	0.013 (0.002 - 0.041)	0.039 (0.012 - 0.081)	0.961 (0.918 - 0.987)	1.112e-05 (3.378e-08 - 0.007)
North-Western	Chavuma	1	76	0	1	0	1	3.931e-08 (3.658e-08 - 5.664e-08)	1 (1 - 1)	1	0	0.983 (0.955 - 0.996)	0.017 (0.004 - 0.045)	0.138 (0.085 - 0.211)	0.862 (0.789 - 0.915)	0
North-Western	Kabompo	1	28	0	1	0	1	0.012 (0 - 0.063)	0.988 (0.937 - 1)	1	0	1 (1 - 1)	3.496e-08 (3.021e-08 - 5.530e-08)	0.039 (0.006 - 0.116)	0.959 (0.881 - 0.994)	1.315e-05 (3.235e-08 - 0.021)
North-Western	Manyinga	1	80	0	1	4.446e-08 (4.446e-08 - 5.361e-08)	1 (1 - 1)	0.018 (0.005 - 0.043)	0.982 (0.957 - 0.995)	1	0	0.955 (0.922 - 0.98)	0.045 (0.02 - 0.078)	0.071 (0.041 - 0.112)	0.914 (0.87 - 0.947)	0.013 (0.003 - 0.035)
North-Western	Mwinilunga	1	38	0	1	0	1	0	1	1 (1 - 1)	4.549e-08 (4.510e-08 - 5.663e-08)	0.975 (0.933 - 0.994)	0.025 (0.006 - 0.067)	1.631e-09 (1.631e-09 - 5.749e-08)	1 (1 - 1)	0
North-Western	Zambezi	1	30	3.563e-08 (3.563e-08 - 4.495e-08)	1 (1 - 1)	0.006 (0 - 0.032)	0.994 (0.968 - 1)	0e0 (0e0 - 3.046e-08)	1 (1 - 1)	1	0	0.994 (0.965 - 1)	0.006 (0 - 0.035)	0.193 (0.104 - 0.311)	0.807 (0.688 - 0.896)	8.777e-06 (2.901e-08 - 0.005)
Southern	Chikankata	1	70	0	1	0	1	0	1	1	0	1 (1 - 1)	0e0 (0e0 - 4.481e-08)	0	1	0
Southern	Chirundu	1	50	0	1	0	1	0	1	1	0	1	0	0	1	0
Southern	Kazungula	1	25	0.044 (0.007 - 0.153)	0.956 (0.847 - 0.993)	0	1	0	1	1	0	0.92 (0.788 - 0.979)	0.08 (0.021 - 0.212)	3.068e-08 (1.181e-12 - 0.012)	0.978 (0.887 - 0.999)	0.021 (0.001 - 0.113)
Southern	Pemba	1	66	0	1	0	1	0	1	1	0	1	0	0	1	0
Southern	Sinazong-we	1	25	4.458e-08 (3.110e-08 - 5.632e-08)	1 (1 - 1)	0	1	0	1	1	0	1	0	0e0 (0e0 - 5.224e-08)	1 (1 - 1)	0
belt	Chilila-bombwe	1	26	0	1	0	1	0	1	1	0	1 (1 - 1)	3.667e-08 (3.610e-08 - 3.900e-08)	0	1	0
	Kalulushi	1	28	0	1	0	1	0	1	1	0	1	0	0	1	0

	Luanshya	1	55	0	1	0	1	0	1	0.994 (0.965 - 1)	0.006 (0 - 0.035)	1	0	0	1	0
	Lufwan-yama	1	29	0	1	0	1	0	1	1	0	1	0	5.113e-08 (3.445e-08 - 5.776e-08)	1 (1 - 1)	0
	Masaiti	1	33	0	1	0	1	0	1	1 (1 - 1)	3.736e-08 (3.648e-08 - 5.411e-08)	1	0	0	1	0
	Mpongwe	1	29	0	1	0	1	0	1	1	0	1	0	0	1	0
	Mufulira	1	64	0	1	0	1	0	1	1	0	1	0	0	1	0
	Ndola	1	27	0	1	0	1	0	1	1	0	1	0	0	1	0
Lusaka	Kafue	1	71	0	1	0	1	0	1	1	0	1	0	0	1	0
	Rufunsa	4	143	0.008 (0.001 - 0.029)	0.992 (0.971 - 0.999)	0	1	0	1	1	0	1	0	0	0.997 (0.982 - 1)	0.003 (1.609e-08 - 0.018)
Central	Chibombo	1	103	0.306 (0.235 - 0.384)	0.694 (0.616 - 0.765)	0	1	0	1	1	0	1	0	0.033 (0.01 - 0.067)	0.967 (0.933 - 0.99)	0
	Chisamba	1	30	0	1	0	1	3.377e-08 (3.134e-08 - 3.901e-08)	1 (1 - 1)	1	0	1	0	0	1	0
	Chitambo	1	24	0	1	0	1	0	1	1	0	1	0	0	1	0
	Kabwe	1	29	0	1	0	1	0	1	1	0	1	0	0	1	0
	Luano	1	27	3.876e-08 (3.846e-08 - 5.688e-08)	1 (1 - 1)	0	1	0	1	1	0	1	0	0	1	0
	Mkushi	1	26	0	1	0	1	0	1	1 (1 - 1)	1.722e-08 (0e0 - 4.662e-08)	1	0	0	1	0
	Serenje	1	73	0	1	0	1	0	1	1 (1 - 1)	4.357e-08 (3.480e-08 - 5.265e-08)	1	0	0	1	0
Luapula	Chembe	1	75	0	1	0	1	0	1	1	0	1	0	0	1	0
	Chifunabuli	1	26	0	1	0	1	0	1	1	0	1	0	0	1	0
	Chipili	1	30	0	1	0	1	0	1	0.993 (0.966 - 1)	0.007 (0 - 0.034)	1	0	0	1	0
	Lunga	1	46	0	1	0	1	0	1	1	0	1	0	0	1	0
	Milenge	1	65	0	1	0	1	0	1	0.997 (0.987 - 1)	0.003 (0 - 0.013)	1	0	0	1	0
	Mwansa-bombwe	1	37	0	1	0	1	0	1	1 (1 - 1)	5.191e-08 (3.786e-08 - 5.191e-08)	1	0	0	1	0
	Mwense	1	29	0	1	0	1	0	1	1 (0.969 - 1)	4.702e-08 (4.337e-08 - 0.031)	1	0	0	1	0

	Nchelenge	1	17	0	1	0	1	0	1	1 (1 - 1)	3.229e-08 (3.229e-08 - 5.479e-08)	1	0	0	1	0
	Samfya	1	25	0	1	0	1	0	1	1	0	1	0	0	1	0
Northern	Luwingu	1	27	0	1	0	1	0	1	1 (1 - 1)	5.627e-08 (3.156e-08 - 5.627e-08)	1	0	0	1	0
	Mbala	1	28	0	1	0	1	0	1	1	0	1	0	0	1	0
	Mporokoso	1	65	0	1	0	1	0	1	1 (1 - 1)	3.279e-08 (3.081e-08 - 5.740e-08)	1	0	0	1	0
	Mpulungu	1	26	0	1	0	1	0	1	1	0	1	0	0	1	0
	Mungwi	1	29	0	1	0	1	0	1	1	0	1	0	0	1	0
	Senga	1	29	0	1	0	1	0	1	1	0	1	0	0	1	0
Muchinga	Kanchibiya	1	47	0	1	0	1	0	1	1 (1 - 1)	4.662e-08 (3.015e-08 - 5.046e-08)	1	0	0	1	0
	Lavushi-manda	1	75	0	1	0	1	0	1	1	0	1	0	0	1	0
	Mafinga	1	25	0	1	0	1	0	1	1	0	1	0	0	1	0
	Nakonde	2	32	0	1	0	1	0	1	1 (1 - 1)	4.334e-08 (4.165e-08 - 5.725e-08)	1	0	0	1	0
	Shiwan-gandu	1	13	0	1	0	1	0	1	1	0	1	0	0	1	0
Eastern	Chipata	1	77	0	1	0	1	0	1	1 (1 - 1)	3.930e-08 (3.247e-08 - 5.362e-08)	1	0	0	1	0
	Katete	1	27	0	1	0	1	0	1	1	0	1	0	0	1	0
	Nyimba	1	29	0	1	0	1	0	1	1	0	1	0	0	1	0
	Petauke	1	18	0	1	0	1	0	1	1	0	1	0	0	1	0
	Sinda	1	26	0	1	0	1	0	1	1 (1 - 1)	4.555e-08 (3.150e-08 - 5.404e-08)	1	0	0	1	0
	Vubwi	1	28	0	1	0	1	0	1	1	0	1	0	0	1	0

[a]: Number of health facilities within district. [b]: number of samples used to estimate allele frequencies

Table S5: Proportion of samples carrying non-synonymous and synonymous *k13* mutations.

k13 Codon	Ref.	Genotype[a]	Overall		Western		North-Western		Southern		Copperbelt		Lusaka		Central		Luapula		Northern		Muchinga		Eastern	
			%	*N*	%	*N*	%	*N*	%	*N*	%	*N*	%	*N*	%	*N*	%	*N*	%	*N*	%	*N*	%	*N*
**441**	P	L	1.25	2314	1.61	186	0	239	0.49	203	0	281	0	199	8.9	281	0	342	0	201	0	188	0	194
		L+P	1.82		6.99		0.42		1.48		0		1.51		7.83		0		0		0		0	
		P	96.93		91.4		99.58		98.03		100		98.49		83.27		100		100		100		100	
**469**	C	C	98.46	2470	100	203	98.41	252	99.57	235	98.31	296	97.66	214	100	310	96.58	351	99.03	206	98.98	197	96.6	206
		C+c	1.17		0		1.19		0.43		1.01		1.4		0		2.85		0.97		1.02		2.43	
		c	0.32		0		0		0		0.68		0.93		0		0.57		0		0		0.97	
		Y	0.04		0		0.4		0		0		0		0		0		0		0		0	
**479**	K	K	99.96	2470	99.51	203	100	252	100	235	100	296	100	214	100	310	100	351	100	206	100	197	100	206
		I	0.04		0.49		0		0		0		0		0		0		0		0		0	
**485**	S	S	99.8	2470	99.01	203	99.21	252	100	235	100	296	100	214	100	310	99.72	351	100	206	100	197	100	206
		N+S	0.08		0		0.4		0		0		0		0		0.28		0		0		0	
		N	0.12		0.99		0.4		0		0		0		0		0		0		0		0	
**504**	A	A	99.56	2292	99.47	188	97.86	234	99.07	214	99.27	275	100	208	100	291	100	321	100	185	100	181	100	195
		A+a	0.39		0.53		2.14		0.93		0.36		0		0		0		0		0		0	
		V	0.04		0		0		0		0.36		0		0		0		0		0		0	
**537**	N	N	99.96	2333	100	190	100	241	100	207	100	282	100	204	100	283	100	343	100	201	99.47	188	100	194
		S	0.04		0		0		0		0		0		0		0		0		0.53		0	
**539**	R	R	99.7	2333	98.95	190	98.76	241	100	207	100	282	99.51	204	100	283	100	343	99.5	201	100	188	100	194
		R+r	0.09		0		0		0		0		0.49		0		0		0.5		0		0	
		I+R	0.13		0		1.24		0		0		0		0		0		0		0		0	
		I	0.09		1.05		0		0		0		0		0		0		0		0		0	
**574**	P	L+P	0.56	2481	2.45	204	3.17	252	0	239	0	297	0	214	0.32	313	0	352	0	206	0	197	0	207
		P	99.36		97.06		96.43		100		100		100		99.68		100		100		100		100	
		L	0.08		0.49		0.4		0		0		0		0		0		0		0		0	
**575**	R	R	99.92	2481	100	204	100	252	100	239	100	297	100	214	100	313	99.43	352	100	206	100	197	100	207
		R+r	0.08		0		0		0		0		0		0		0.57		0		0		0	
**578**	A	A	99.11	2481	100	204	99.6	252	100	239	98.99	297	99.53	214	99.04	313	97.73	352	99.03	206	98.98	197	99.03	207
		A+S	0.81		0		0.4		0		1.01		0.47		0.64		1.99		0.97		1.02		0.97	
		S	0.08		0		0		0		0		0		0.32		0.28		0		0		0	
**599**	N	N	99.96	2411	100	200	100	247	99.57	234	100	288	100	212	100	305	100	334	100	196	100	192	100	203
		N+n	0.04		0		0		0.43		0		0		0		0		0		0		0	
**605**	E	E	99.88	2411	100	200	100	247	99.57	234	100	288	100	212	100	305	100	334	100	196	100	192	99.01	203
		E+e	0.12		0	0	0	0	0.43	234	0	0	0	0	0	0	0	0	0	0	0	0	0.99	
**613**	Q	Q	99.63	2411	99	200	98.79	247	99.57	234	100	288	100	212	100	305	99.7	334	99.49	196	99.48	192	100	203
		E+Q	0.17		0		0.4		0		0		0		0		0.3		0.51		0.52		0	
		Q+q	0.17		0.5		0.81		0.43		0		0		0		0		0		0		0	
		q	0.04		0.5		0		0		0		0		0		0		0		0		0	
**622**	R	R	98.48	2168	96.34	164	90.62	224	97.27	183	99.63	270	100	189	100	253	100	326	100	196	100	181	100	182
		R+T	1.2		2.44		8.04		1.64		0.37		0		0		0		0		0		0	
		T	0.32		1.22		1.34		1.09		0		0		0		0		0		0		0	

**667**	P	A	0.83	2168	3.66	164	4.02	224	1.09	183	0	270	0	189	0.4	253	0	326	0	196	0	181	0	182
		A+P	2.58		5.49		18.3		0		0.37		0		1.98		0		0		0		0	
		P	96.08		90.24		75		97.81		99.63		98.94		97.63		100		100		100		100	
		S	0.14		0		0		0.55		0		1.06		0		0		0		0		0	
		A+P+S	0.05		0		0.45		0		0		0		0		0		0		0		0	
		P+S	0.32		0.61		2.23		0.55		0		0		0		0		0		0		0	
**675**	A	A	99.91	2168	100	164	99.55	224	99.45	183	100	270	100	189	100	253	100	326	100	196	100	181	100	182
		A+V	0.05		0		0.45		0		0		0		0		0		0		0		0	
		V	0.05		0		0		0.55		0		0		0		0		0		0		0	
**690**	G	G	99.77	2168	100	164	100	224	100	183	100	270	100	189	99.6	253	99.69	326	98.47	196	100	181	100	182
		G+g	0.14		0		0		0		0		0		0.4		0.31		0.51		0		0	
		g	0.09		0		0		0		0		0		0		0		1.02		0		0	

[a]Synonymous mutations denoted with lowercase

Table S6: Adjusted prevalence for mutations observed in >0.5% of the samples. Source data for Figures 2B and 3B.

	Estimated prevalence of infections carrying a mutation (95% CI)											Design effect	Intraclass Correlation Coefficient
SNP	Country	Central	Copperbelt	Eastern	Luapula	Lusaka	Muchinga	North Western	Northern	Southern	Western		
k13 441L	1.39% (0.45-3.25%)	5.32% (0.03-33.8%)	0%	0%	0%	1.98% (0.1-9.11%)	0%	0.82% (0-7%)	0%	2.11% (0-24.92%)	3.69% (0-32.67%)	3.77	0.08
k13 539I	0.46% (0.13-1.15%)	0%	0%	0%	0%	0%	0%	2.06% (0.06-10.79%)	0%	0%	2.49% (0.48-7.32%)	5.94	0.13
k13 574L	0.66% (0.27-1.34%)	0.41% (0-3.41%)	0%	0%	0%	0%	0%	3.84% (0.96-10.01%)	0%	0%	2.36% (0.06-12.3%)	4.69	0.09
k13 578S	0.88% (0.5-1.43%)	1.48% (0.28-4.42%)	0.41% (0.01-2.16%)	0.65% (0.05-2.66%)	3.15% (1.25-6.47%)	1.05% (0-8.25%)	0.88% (0.04-4.26%)	0.6% (0-5.36%)	0.55% (0.01-3.27%)	0%	0%	1.94	0.02
k13 622T	1.54% (0.73-2.85%)	0%	0.61% (0-4.34%)	0%	0%	0%	0%	9.37% (5.58-14.53%)	0%	2.75% (0-32%)	2.69% (0.03-16.02%)	4.90	0.11
k13 667A	3.65% (1.8-6.53%)	0.68% (0-5.8%)	0.54% (0-3.81%)	0%	0%	0%	0%	21.63% (4.2-52.91%)	0%	0.77% (0-9%)	12.91% (7.09-21.01%)	20.34	0.56
k13 667S	0.51% (0.19-1.09%)	0%	0%	0%	0%	0.37% (0-4.26%)	0%	2.64% (0.78-6.36%)	0%	1.34% (0-17.28%)	0.74% (0-6.17%)	2.53	0.04
dhfr 164L	0.16% (0.01-0.85%)	0%	0%	0%	0%	0%	1.63% (0.05-8.45%)	0%	0%	0%	0%	NA	NA
dhfr 51I	97.77% (91.76-99.77%)	99.05% (97.31-99.79%)	98.99% (94.57-99.97%)	99.13% (93.91-99.99%)	100%	100%	99.96% (99.52-100%)	100%	99.28% (93.29-100%)	81.59% (64.42-92.82%)	99.65% (95.13-100%)	8.43	0.20
dhfr 59R	96.65% (89.43-99.47%)	99.12% (97.22-99.85%)	99.37% (97.52-99.94%)	99.22% (93.28-100%)	99.37% (97.82-99.91%)	99.78% (98.1-100%)	99.49% (96.41-100%)	98% (91.36-99.88%)	98.19% (94.2-99.71%)	76.97% (50.79-93.41%)	96.99% (77.76-99.99%)	8.00	0.19
dhps 436A	4.17% (2.8-5.96%)	2.9% (0.73-7.53%)	0.24% (0-1.95%)	0%	4.81% (2.16-9.12%)	0.16% (0-2.06%)	1.26% (0.04-6.57%)	18.87% (11.02-29.12%)	1.82% (0.08-8.59%)	1.12% (0-14.72%)	10.52% (7.48-14.27%)	5.34	0.11
dhps 436F	0.06% (0-0.28%)	0%	0.51% (0-3.49%)	0%	0%	0.05% (0-0.69%)	0%	0%	0%	0%	0%	2.75	0.05
dhps 437G	97.62% (95.37-98.96%)	96.99% (93.17-99.01%)	98.42% (95.21-99.71%)	99.13% (93.91-99.99%)	97.11% (91.61-99.43%)	98.92% (92.96-99.99%)	99.2% (95.76-99.98%)	99.58% (97.04-100%)	97.01% (90.58-99.52%)	90.78% (79.42-97.04%)	99.04% (94.11-99.99%)	2.62	0.04
dhps 540E	93.26% (91.59-94.69%)	95.69% (90.11-98.62%)	90.49% (83.64-95.15%)	98.73% (95.57-99.84%)	94.96% (90.28-97.81%)	96.68% (90.85-99.26%)	98.32% (89.23-99.98%)	87.38% (77.13-94.18%)	95.19% (85.58-99.17%)	88.91% (83.99-92.74%)	86.28% (82.42-89.56%)	3.08	0.06
dhps 581G	4.99% (3.44-6.98%)	3.1% (0.86-7.71%)	7.02% (3.85-11.59%)	2.05% (0.52-5.34%)	12.87% (5.29-24.85%)	0.16% (0-1.84%)	2.97% (0.2-12.29%)	3.79% (1.78-6.99%)	8.52% (1.8-22.88%)	1.14% (0-11.22%)	8.34% (0.56-31.79%)	3.90	0.07

dhps 613S	0.07% (0.01-0.25%)	0%	0.26% (0-1.78%)	0%	0%	0.05% (0-0.66%)	0%	0.42% (0-2.95%)	0%	0%	0%	1.97	0.03
dhfr 108N	100%	100%	100%	100%	100%	100%	100%	100%	100%	100%	100%	NA	NA
PF3D7_1322700 236I	0.4% (0.13-0.93%)	0.5% (0-4.31%)	0%	0.37% (0-3.61%)	0%	0%	0.44% (0-4.19%)	0.91% (0-7.75%)	1.73% (0.12-7.25%)	0%	0%	3.28	0.07
mdr1 1246Y	1.15% (0.65-1.89%)	0.61% (0.03-2.75%)	0.24% (0-1.95%)	0%	0.3% (0.02-1.34%)	0%	0.5% (0-3.82%)	1.64% (0-13.65%)	0%	4.2% (2.57-6.45%)	4.05% (2.04-7.13%)	5.08	0.11
mdr1 184F	50.08% (42.63-57.53%)	45.82% (36.15-55.73%)	52.15% (45.71-58.55%)	48.42% (33.58-63.47%)	68.07% (57.52-77.41%)	48.43% (27.36-69.92%)	56.94% (50.05-63.64%)	58.4% (48.9-67.46%)	53.98% (36.47-70.8%)	16.19% (0.63-59.36%)	52.4% (46.52-58.24%)	4.02	0.08
mdr2 492V	49.43% (45.49-53.37%)	54.76% (43.72-65.47%)	51.7% (42.34-60.97%)	34.73% (21.29-50.23%)	65.84% (58.11-73%)	41.91% (27.5-57.4%)	40.28% (24.88-57.23%)	64.61% (48.17-78.85%)	49.05% (42.9-55.22%)	38.84% (29.74-48.55%)	52.56% (24.81-79.19%)	3.79	0.08
mdr1 86Y	0.13% (0.03-0.36%)	0%	0.35% (0-2.39%)	0%	0.91% (0.11-3.25%)	0%	0%	0%	0%	0%	0.09% (0-1.67%)	1.31	0.01
crt CVMNK 72-76 CVIET	0.18% (0.04-0.5%)	0.37% (0-3.17%)	0.51% (0-3.5%)	0%	0.24% (0-1.78%)	0%	0%	0%	0%	0%	0.71% (0-5.98%)	1.96	0.02
crt 48L	0.34% (0.11-0.81%)	0%	0.53% (0.04-2.26%)	0%	0%	0%	0%	0%	1.01% (0.01-6.54%)	1.15% (0.37-2.68%)	0.71% (0-5.98%)	1.79	0.02

Table S7: Multivariate logistic regression of select demographic characteristics and genotypes.

	k13 P441L (66/2031)[a]			*k13* R622T (22/1957)[a]			*k13* P667S (60/1957)[a]			*k13* P441L, C469Y, K479I, P574L, R622T, P667S or A675V (154/2195)[a]			*dhps* S436A (87/2135)[a]			*dhps* K540E (1856/2016)[a]			*dhps* A581G (107/2187)[a]		
	n[b]/*N*[c]	OR[d] (95%CI[e])	*P*-value	*n*[b]/*N*[c]	OR[d] (95%CI[e])	p value	*n*[b]/*N*[c]	OR[d] (95%CI[e])	*P*-value	*n*[b]/*N*[c]	OR[d] (95%CI[e])	*P*-value	*n*[b]/*N*[c]	OR[d] (95%CI[e])	*P*-value	*n*[b]/*N*[c]	OR[d] (95%CI[e])	*P*-value	*n*[b]/*N*[c]	OR[d] (95%CI[e])	*P*-value
**Age**																					
**< 5 years**	5/223	Ref.		1/213	Ref.		6/213	Ref.		16/236	Ref.		10/228	Ref.		208/221	Ref.		13/236	Ref.	
**5 - 14 years**	10/783	0.36 (0.10, 1.37)	0.14	11/733	3.78 (0.46, 31.2)	0.2	27/733	0.96 (0.33, 2.79)	>0.9	50/854	0.67 (0.33, 1.38)	0.3	37/831	0.83 (0.38, 1.81)	0.6	720/782	0.85 (0.45, 1.60)	0.6	47/853	1.01 (0.51, 2.01)	>0.9
**15 - 24 years**	23/555	0.72 (0.18, 2.91)	0.6	8/534	7.6 (0.69, 83.2)	0.1	17/534	0.78 (0.20, 3.01)	0.7	52/623	0.99 (0.42, 2.33)	>0.9	25/606	0.63 (0.23, 1.70)	0.4	520/571	0.82 (0.39, 1.75)	0.6	24/618	0.64 (0.26, 1.59)	0.3
**≥25 years**	28/419	1.33 (0.30, 6.00)	0.7	2/412	2.13 (0.11, 39.6)	0.6	7/412	0.6 (0.12, 3.06)	0.5	36/482	0.98 (0.36, 2.66)	>0.9	17/470	0.63 (0.20, 1.99)	0.4	408/442	0.85 (0.36, 2.02)	0.7	23/480	0.98 (0.35, 2.75)	>0.9
**Occupation**																					
**Minor**	11/894	Ref.		12/831	Ref.		30/831	Ref.		61/964	Ref.		42/939	Ref.		892/827	Ref.		50/963	Ref.	
**Student**	8/264	**5.41 (1.37, 21.3)**	**0.016**	2/253	0.31 (0.05, 1.90)	0.2	7/253	2.17 (0.63, 7.49)	0.2	18/294	1.68 (0.74, 3.77)	0.2	12/279	1.9 (0.79, 4.54)	0.2	262/238	0.93 (0.51, 1.71)	0.8	16/293	1.27 (0.60, 2.67)	0.5
**Unem-ployed**	31/319	1.03 (0.32, 3.32)	>0.9	4/295	0.87 (0.19, 3.94)	0.9	13/295	1.32 (0.43, 4.00)	0.6	46/340	0.84 (0.41, 1.72)	0.6	15/335	1.02 (0.42, 2.52)	>0.9	317/286	0.8 (0.42, 1.53)	0.5	17/337	1.32 (0.57, 3.08)	0.5
**Agricultural**	13/383	0.85 (0.22, 3.33)	0.8	3/348	1.11 (0.14, 8.78)	>0.9	5/348	1.32 (0.27, 6.46)	0.7	21/418	0.79 (0.32, 1.96)	0.6	15/405	1.8 (0.65, 5.01)	0.3	376/350	0.97 (0.47, 2.00)	>0.9	14/417	0.69 (0.26, 1.81)	0.5
**Other**	3/171	0.58 (0.09, 3.87)	0.6	1/165	0.22 (0.02, 2.45)	0.2	2/165	0.85 (0.18, 4.09)	0.8	8/179	0.61 (0.21, 1.73)	0.4	5/177	1.11 (0.34, 3.60)	0.9	169/155	0.88 (0.41, 1.91)	0.8	10/177	1.19 (0.45, 3.15)	0.7
**Sex**																					
**Female**	29/995	Ref.		11/926	Ref.		38/926	Ref.		79/1073	Ref.		37/1046	Ref.		985/898	Ref.		54/1067	Ref.	
**Male**	37/1036	1.38 (0.73, 2.63)	0.3	11/966	1.27 (0.52, 3.11)	0.6	19/966	0.59 (0.31, 1.10)	0.1	75/1122	1.06 (0.71, 1.58)	0.8	**52/1089**	**1.62 (1.02, 2.57)**	**0.042**	1031/958	1.27 (0.91, 1.76)	0.2	53/1120	0.93 (0.62, 1.39)	0.7
**Travel History**																					
**No travel**	62/1976	Ref.		NA	NA	NA	NA	NA	NA	150/2137	Ref.		NA	NA	NA	1807/1961	Ref.		103/2130	Ref.	
**Travel**	4/55	**10.1 (1.58, 64.5)**	**0.015**	NA	NA	NA	NA	NA	NA	4/58	3.33 (0.82, 13.5)	0.091	NA	NA	NA	49/55	0.77 (0.31, 1.90)	0.6	4/57	1.85 (0.62, 5.51)	0.3

[a]total samples with the mutation/total samples. [b]samples with the mutation within category. [c]total observations within category. [d]OR: odds ratio. [e]CI: confidence interval

Table S8: Non-synonymous mutations not reported in Table 2.

Gene	Observed in >0.5% of the samples	Observed in ≤0.5% of the samples
arps10	None	None
coronin	None	None
exo	None	E415G
fd	None	None
mdr2	I492V	None
PF3D7_1322700	None	T236I
pib7	None	C1484F

Table S9: Proportion of samples carrying a non-synonymous or synonymous mutation in *crt*, *exo*, *mdr2*, PF3D7_1322700 and *pib7*.

				**Overall**		**Western**		**North-Western**		**Southern**		**Copperbelt**		**Lusaka**		**Central**		**Luapula**		**Northern**		**Muchinga**		**Eastern**	
**Gene**	**Codon**	**Ref.**	**Genotype[a]**	**%**	***N***	**%**	***N***	**%**	***N***	**%**	***N***	**%**	***N***	**%**	***N***	**%**	***N***	**%**	***N***	**%**	***N***	**%**	***N***	**%**	***N***
crt	48	F	F	99.8	2480	99.51	204	100	252	99.58	237	99.33	297	100	214	100	313	100	353	99.51	206	100	197	100	207
			F+L	0.2		0.49		0		0.42		0.67		0		0		0		0.49		0		0	
	72/73/74/75/76	CVMNK	CVIET+CVMNK	0.16	2486	0.49	204	0	252	0	241	0.67	297	0	214	0.32	314	0	354	0	206	0	197	0	207
			CVMNK	99.72		99.51		99.6		100		99.33		100		99.68		99.44		100		100		100	
			CVMNK+CVIET	0.04		0		0		0		0		0		0		0.28		0		0		0	
			CVMNK+YVMNK	0.08		0		0.4		0		0		0		0		0.28		0		0		0	
	218/220	IA	IA	99.9	1015	100	70	99.04	104	100	81	100	116	100	77	100	115	100	182	100	81	100	81	100	108
			IA+IS	0.1		0		0.96	104	0		0		0		0		0		0		0		0	
	343/350/353/356	MCGI	MCGI	99.91	2311	100	197	100	239	100	219	99.63	269	99.51	205	100	295	100	322	100	186	100	181	100	198
			MCGI+MCGT	0.09		0		0		0		0.37		0.49		0		0		0		0		0	
exo	415	E	E	99.78	2282	100	184	100	231	100	202	100	276	99.49	197	100	280	100	340	99.49	197	98.37	184	100	191
			e	0.09		0		0		0		0		0.51		0		0		0		0.54		0	
			E+e	0.09		0		0		0		0		0		0		0		0.51		0.54		0	
			G	0.04		0		0		0		0		0		0		0		0		0.54		0	
mdr2	463/484	DT	DT	99.91	2204	100	174	99.56	225	100	191	99.63	272	100	190	100	259	100	333	100	192	100	183	100	185
			DT+Dt	0.09		0		0.44		0		0.37		0		0		0		0		0		0	
	492	I	I	50.32	2200	50.57	174	37.5	224	61.78	191	47.23	271	65.08	189	45.56	259	36.64	333	47.4	192	59.56	183	68.48	184
			I+V	31.23		33.33		45.98		20.42		31.73		14.29		27.41		48.35		33.85		27.87		14.13	
			V	18.45		16.09		16.52		17.8		21.03		20.63		27.03		15.02		18.75		12.57		17.39	
PF3D7_1322700	236	T	I	0.15	2030	0	154	0	209	0	191	0	238	0	162	0.4	248	0	314	0	177	0.58	171	0.6	166
			T	99.7		100		99.52		100		100		100		99.6		100		98.87		99.42		99.4	
			I+T	0.15		0		0.48		0		0		0		0		0		1.13		0		0	
pib7	1484	C	C	99.96	2327	99.47	189	100	240	100	208	100	281	100	200	100	283	100	343	100	201	100	186	100	196
			F	0.04		0.53		0		0		0		0		0		0		0		0		0	

[a]Synonymous mutations denoted with lowercase

Table S10: District-level *dhfr* microhaplotype allele frequencies. Source data for Figure 3A.

Allele frequencies (95% confidence interval)

Province	District	HFs[a]	Samples[b]	*dhfr* 16/51/59				*dhfr* 108/164		
				AIC	AIR	ANR	ANC	NI	SI	NL
Western	Kalabo	1	27	0.055 (0.034 - 0.084)	0.918 (0.887 - 0.944)	0.025 (0.012 - 0.043)	0	1	0	0
	Sesheke	1	29	0.027 (0.017 - 0.042)	0.957 (0.94 - 0.97)	0.016 (0.008 - 0.027)	0	1	0	0
	Shangombo	1	87	0.062 (0.04 - 0.086)	0.888 (0.857 - 0.918)	0.049 (0.032 - 0.073)	0	1	0	0
	Sikongo	1	61	0.022 (0.012 - 0.036)	0.963 (0.946 - 0.976)	0.014 (0.007 - 0.025)	0	1 (1 - 1)	3.095e-08 (3.095e-08 - 5.514e-08)	0
North-Western	Chavuma	1	76	0.014 (0.004 - 0.03)	0.98 (0.961 - 0.993)	0.006 (0.001 - 0.019)	0	1	0	0
	Kabompo	1	28	0.017 (0.008 - 0.03)	0.951 (0.93 - 0.967)	0.032 (0.019 - 0.051)	0	1	0	0
	Manyinga	1	80	0.044 (0.033 - 0.058)	0.94 (0.924 - 0.953)	0.014 (0.008 - 0.022)	0.001 (0 - 0.005)	0.999 (0.995 - 1)	0.001 (0 - 0.005)	0
	Mwinilunga	1	38	0.041 (0.024 - 0.065)	0.94 (0.911 - 0.96)	0.019 (0.008 - 0.036)	0	1	0	0
	Zambezi	1	30	0.035 (0.021 - 0.055)	0.953 (0.93 - 0.97)	0.011 (0.004 - 0.023)	2.157e-08 (1.742e-12 - 0.001)	0.998 (0.992 - 1)	0	0.002 (4.514e-05 - 0.008)
Southern	Chikankata	1	70	0.002 (3.595e-08 - 0.013)	0.998 (0.987 - 1)	7.621e-11 (1.054e-12 - 0)	0	1	0	0
	Chirundu	1	50	0.016 (0 - 0.094)	0.983 (0.899 - 0.999)	1.576e-05 (3.508e-08 - 0.021)	0	1	0	0
	Kazungula	1	25	1.170e-05 (3.671e-08 - 0.014)	1 (0.982 - 1)	7.754e-06 (3.541e-08 - 0.01)	0	1	0	0
	Pemba	1	66	0.056 (0.026 - 0.106)	0.894 (0.833 - 0.938)	0.047 (0.02 - 0.092)	0	1	0	0
	Sinazongwe	1	25	0.052 (0.031 - 0.096)	0.948 (0.904 - 0.969)	1.985e-12 (1.029e-12 - 3.136e-11)	0	1	0	0
Copperbelt	Chililabombwe	1	26	0.013 (0.003 - 0.033)	0.968 (0.939 - 0.986)	0.018 (0.006 - 0.041)	0	1	0	0
	Kalulushi	1	28	0.037 (0.006 - 0.109)	0.963 (0.891 - 0.994)	1.717e-12 (1.028e-12 - 2.647e-11)	0	1	0	0
	Luanshya	1	55	0.032 (0.013 - 0.062)	0.958 (0.925 - 0.98)	0.008 (0.001 - 0.028)	0	1	0	0
	Lufwanyama	1	29	9.944e-06 (3.443e-08 - 0.008)	0.993 (0.963 - 1)	0.006 (0 - 0.035)	0	1	0	0
	Masaiti	1	33	0.018 (0.002 - 0.058)	0.981 (0.941 - 0.997)	9.855e-06 (3.293e-08 - 0.007)	0	1	0	0
	Mpongwe	1	29	0	0.997 (0.985 - 1)	0.003 (0 - 0.015)	0	1	0	0

	Mufulira	1	64	5.113e-08 (0e0 - 5.616e-08)	1 (1 - 1)	0	0	1	0	0
	Ndola	1	27	0	1 (1 - 1)	4.378e-09 (0e0 - 5.779e-08)	0	1	0	0
Lusaka	Kafue	1	71	0.385 (0.266 - 0.512)	0.359 (0.25 - 0.478)	0.251 (0.155 - 0.368)	0	1	0	0
	Rufunsa	4	143	0.026 (0.001 - 0.129)	0.941 (0.805 - 0.991)	0.022 (0.001 - 0.13)	0	1	0	0
Central	Chibombo	1	103	0	1 (1 - 1)	4.398e-08 (4.034e-08 - 5.799e-08)	0	1	0	0
	Chisamba	1	30	7.389e-09 (3.659e-12 - 1.324e-08)	1 (1 - 1)	4.931e-08 (3.087e-08 - 4.931e-08)	0	1	0	0
	Chitambo	1	24	0.016 (0.003 - 0.05)	0.983 (0.95 - 0.997)	1.132e-05 (3.501e-08 - 0.006)	0	1	0	0
	Kabwe	1	29	5.363e-08 (3.086e-08 - 5.471e-08)	1 (1 - 1)	0	0	1	0	0
	Luano	1	27	0.022 (0.005 - 0.059)	0.955 (0.907 - 0.982)	0.021 (0.005 - 0.053)	0	1	0	0
	Mkushi	1	26	0	1 (1 - 1)	0e0 (0e0 - 5.535e-08)	0	1	0	0
	Serenje	1	73	0.026 (0.008 - 0.064)	0.967 (0.926 - 0.988)	0.005 (0 - 0.024)	0	1 (1 - 1)	5.055e-08 (2.988e-08 - 5.260e-08)	0
Luapula	Chembe	1	75	4.465e-08 (3.785e-08 - 5.892e-08)	1 (1 - 1)	0	0	1	0	0
	Chifunabuli	1	26	0.012 (0 - 0.056)	0.988 (0.944 - 1)	0	0	1	0	0
	Chipili	1	30	0.004 (0 - 0.018)	0.987 (0.966 - 0.997)	0.008 (0.001 - 0.025)	0	1	0	0
	Lunga	1	46	8.284e-06 (3.516e-08 - 0.004)	0.959 (0.923 - 0.982)	0.04 (0.018 - 0.077)	0	1	0	0
	Milenge	1	65	4.455e-08 (4.032e-08 - 5.480e-08)	1 (1 - 1)	0	0	1	0	0
	Mwansa bombwe	1	37	2.965e-12 (1.074e-12 - 0.009)	0.984 (0.853 - 1)	0.015 (3.536e-08 - 0.144)	0	1	0	0
	Mwense	1	29	3.704e-08 (2.993e-08 - 5.103e-08)	1 (1 - 1)	0	0	1	0	0
	Nchelenge	1	17	0	1	0	0	1	0	0
	Samfya	1	25	1.309e-05 (3.334e-08 - 0.013)	0.962 (0.865 - 0.994)	0.037 (0.006 - 0.133)	0	1	0	0
Northern	Luwingu	1	27	0.026 (0.009 - 0.061)	0.948 (0.904 - 0.974)	0.024 (0.008 - 0.058)	0	1	0	0
	Mbala	1	28	0.064 (0.03 - 0.085)	0.928 (0.902 - 0.963)	0.008 (0.001 - 0.025)	1.664e-12 (1.010e-12 - 2.212e-11)	1 (1 - 1)	5.398e-08 (3.177e-08 - 5.700e-08)	0
	Mporokoso	1	65	0.023 (0.004 - 0.07)	0.963 (0.908 - 0.992)	0.009 (0 - 0.046)	0	1	0	0
	Mpulungu	1	26	0.016 (0.002 - 0.054)	0.974 (0.93 - 0.993)	0.006 (0 - 0.035)	1.091e-05 (3.378e-08 - 0.007)	1 (1 - 1)	5.579e-08 (3.845e-08 - 5.755e-08)	0
	Mungwi	1	29	0.051 (0.018 - 0.104)	0.949 (0.896 - 0.982)	1.878e-12 (1.021e-12 - 3.828e-11)	0	1	0	0
	Senga	1	29	0.046 (0.024 - 0.08)	0.943 (0.906 - 0.968)	0.01 (0.003 - 0.026)	0	1	0	0

Muchinga	Kanchibiya	1	47	0.023 (0.006 - 0.053)	0.976 (0.946 - 0.994)	4.651e-06 (3.121e-08 - 0.006)	0	1	0	0
	Lavushimanda	1	75	0.009 (0 - 0.045)	0.979 (0.932 - 0.997)	0.008 (0 - 0.043)	6.920e-06 (3.433e-08 - 0.008)	1 (1 - 1)	5.247e-08 (3.169e-08 - 5.647e-08)	0
	Mafinga	1	25	3.513e-08 (3.513e-08 - 5.412e-08)	1 (1 - 1)	0	0	1	0	0
	Nakonde	2	32	2.528e-08 (1.493e-12 - 0.005)	0.986 (0.93 - 0.999)	0.013 (0 - 0.067)	0	1	0	0
	Shiwan-gandu	1	13	0.052 (0.012 - 0.144)	0.872 (0.756 - 0.943)	0.071 (0.025 - 0.172)	0	1	0	0
Eastern	Chipata	1	77	0.039 (0.006 - 0.122)	0.961 (0.878 - 0.994)	0	0	1	0	0
	Katete	1	27	0.022 (0.006 - 0.06)	0.959 (0.915 - 0.985)	0.015 (0.002 - 0.047)	0	1	0	0
	Nyimba	1	29	4.146e-08 (3.225e-08 - 5.538e-08)	1 (1 - 1)	0	0	1	0	0
	Petauke	1	18	0.029 (0.004 - 0.092)	0.971 (0.908 - 0.996)	0	0	1	0	0
	Sinda	1	26	0.012 (0 - 0.056)	0.986 (0.941 - 1)	1.325e-05 (3.849e-08 - 0.013)	0	1	0	0
	Vubwi	1	28	0.025 (0.008 - 0.057)	0.949 (0.908 - 0.977)	0.024 (0.007 - 0.055)	0	1	0	0

[a]: Number of health facilities within district. [b]: number of samples used to estimate allele frequencies

Table S11: District-level *dhps* microhaplotype allele frequencies. Source data for Figure 3A.

Allele frequencies (95% confidence interval)

Province	District	HFs[a]	Samples[b]	*dhps* 431/436/437						*dhps* 540/581			*dhps* 613	
				IAA	IAG	ISA	ISG	IFA	IFG	EA	EG	KA	A	S
Western	Kalabo	1	27	6.171e-06 (3.014e-08 - 0.002)	0.034 (0.018 - 0.053)	0.047 (0.028 - 0.071)	0.918 (0.89 - 0.943)	0	0	0.698 (0.644 - 0.746)	0.046 (0.028 - 0.072)	0.255 (0.21 - 0.306)	1	0
	Sesheke	1	29	0.008 (0.003 - 0.016)	0.058 (0.042 - 0.078)	0.014 (0.007 - 0.024)	0.919 (0.898 - 0.938)	0	0	0.649 (0.597 - 0.696)	0.014 (0.006 - 0.025)	0.337 (0.29 - 0.388)	1 (1 - 1)	4.745e-08 (3.411e-08 - 5.000e-08)
	Shangombo	1	87	0	0.002 (0 - 0.008)	0.047 (0.03 - 0.07)	0.951 (0.927 - 0.968)	0	0	0.749 (0.698 - 0.792)	0.03 (0.016 - 0.049)	0.22 (0.182 - 0.269)	1	0
	Sikongo	1	61	3.131e-08 (2.465e-12 - 0.001)	0	0.061 (0.044 - 0.081)	0.937 (0.917 - 0.954)	0.001 (2.910e-05 - 0.006)	0	0.816 (0.78 - 0.845)	0.031 (0.019 - 0.05)	0.151 (0.126 - 0.186)	1 (1 - 1)	5.277e-08 (3.441e-08 - 5.948e-08)
North-Western	Chavuma	1	76	0	0.006 (0.001 - 0.017)	0.032 (0.016 - 0.054)	0.961 (0.937 - 0.979)	0	7.572e-06 (3.586e-08 - 0.003)	0.883 (0.838 - 0.919)	6.007e-11 (1.050e-12 - 0.001)	0.117 (0.081 - 0.162)	1 (1 - 1)	3.447e-08 (3.272e-08 - 5.855e-08)
	Kabompo	1	28	0.001 (4.584e-05 - 0.006)	0.018 (0.009 - 0.032)	0.059 (0.04 - 0.08)	0.921 (0.897 - 0.942)	0	0	0.866 (0.837 - 0.895)	0.011 (0.004 - 0.023)	0.122 (0.094 - 0.15)	1	0
	Manyinga	1	80	0.007 (0.004 - 0.014)	0.004 (0.001 - 0.009)	0.111 (0.092 - 0.132)	0.877 (0.855 - 0.896)	0	0	0.773 (0.741 - 0.802)	0.026 (0.017 - 0.036)	0.202 (0.172 - 0.232)	1	0
	Mwinilunga	1	38	0.004 (0.001 - 0.014)	0.004 (0.001 - 0.013)	0.08 (0.056 - 0.111)	0.91 (0.881 - 0.935)	0	0	0.834 (0.793 - 0.87)	0.045 (0.029 - 0.071)	0.119 (0.088 - 0.155)	1	0
	Zambezi	1	30	0.004 (0.001 - 0.014)	0	0.069 (0.045 - 0.096)	0.927 (0.898 - 0.951)	0	0	0.904 (0.875 - 0.931)	0.004 (0.001 - 0.013)	0.091 (0.065 - 0.122)	1	0
Southern	Chikankata	1	70	0	0	0.012 (0.003 - 0.026)	0.988 (0.974 - 0.997)	0	0	0.975 (0.955 - 0.989)	0.005 (0.001 - 0.017)	0.018 (0.007 - 0.037)	1	0
	Chirundu	1	50	0	0.042 (0.006 - 0.119)	0.014 (0 - 0.077)	0.935 (0.844 - 0.987)	0	0	0.713 (0.564 - 0.843)	0.016 (0 - 0.082)	0.262 (0.132 - 0.414)	1	0
	Kazungula	1	25	0	0.011 (0 - 0.062)	0	0.989 (0.938 - 1)	0	0	0.688 (0.537 - 0.805)	0.223 (0.125 - 0.362)	0.086 (0.031 - 0.172)	1	0
	Pemba	1	66	0	0.012 (0.002 - 0.037)	0.094 (0.051 - 0.152)	0.892 (0.828 - 0.936)	0	0	0.705 (0.613 - 0.789)	0.005 (0 - 0.025)	0.287 (0.205 - 0.381)	1	0
	Sinazongwe	1	25	7.119e-06 (3.478e-08 - 0.005)	0.037 (0.014 - 0.084)	0.014 (0.002 - 0.042)	0.946 (0.898 - 0.976)	0	0	0.696 (0.598 - 0.784)	0.005 (0 - 0.03)	0.296 (0.208 - 0.396)	1	0

Copperbelt	Chililabombwe	1	26	4.326e-06 (3.081e-08 - 0.003)	0.07 (0.039 - 0.113)	6.572e-06 (3.179e-08 - 0.003)	0.93 (0.887 - 0.961)	0	0	0.592 (0.497 - 0.676)	0.023 (0.009 - 0.054)	0.383 (0.297 - 0.474)	1	0
	Kalulushi	1	28	0	0.028 (0.004 - 0.097)	1.772e-05 (3.645e-08 - 0.012)	0.97 (0.902 - 0.995)	0	0	0.729 (0.563 - 0.847)	0	0.271 (0.153 - 0.437)	1	0
	Luanshya	1	55	0.008 (0.001 - 0.028)	0.033 (0.013 - 0.063)	0.034 (0.015 - 0.064)	0.921 (0.878 - 0.954)	0	0	0.639 (0.556 - 0.72)	0.008 (0.001 - 0.026)	0.35 (0.272 - 0.433)	1 (1 - 1)	4.067e-08 (4.067e-08 - 5.086e-08)
	Lufwanyama	1	29	0.015 (0.002 - 0.048)	0.034 (0.011 - 0.082)	1.102e-05 (3.384e-08 - 0.007)	0.948 (0.895 - 0.979)	0	0	0.644 (0.57 - 0.752)	2.037e-12 (1.020e-12 - 3.350e-11)	0.356 (0.248 - 0.43)	1	0
	Masaiti	1	33	0	0.075 (0.031 - 0.144)	0	0.925 (0.856 - 0.969)	0	0	0.759 (0.63 - 0.869)	1.851e-12 (1.014e-12 - 3.014e-11)	0.241 (0.131 - 0.37)	1	0
	Mpongwe	1	29	0	0	0.023 (0.009 - 0.047)	0.977 (0.953 - 0.991)	0	0	0.735 (0.665 - 0.803)	0.048 (0.025 - 0.083)	0.215 (0.152 - 0.281)	1	0
	Mufulira	1	64	0	0	0e0 (0e0 - 2.583e-08)	1 (1 - 1)	0	0	0.604 (0.477 - 0.727)	0	0.396 (0.273 - 0.523)	1	0
	Ndola	1	27	0	0.016 (0.001 - 0.086)	0	0.984 (0.914 - 0.999)	0	0	0.847 (0.701 - 0.939)	2.219e-05 (2.788e-08 - 0.018)	0.152 (0.059 - 0.299)	1	0
Lusaka	Kafue	1	71	0	0	0.134 (0.073 - 0.22)	0.866 (0.78 - 0.927)	0	0	0.916 (0.821 - 0.967)	0	0.084 (0.033 - 0.179)	1	0
	Rufunsa	4	143	0	0	0.023 (0.001 - 0.117)	0.977 (0.883 - 0.999)	0	0	0.906 (0.773 - 0.978)	0	0.094 (0.022 - 0.227)	1	0
Central	Chibombo	1	103	0	0	0.122 (0.041 - 0.238)	0.878 (0.762 - 0.959)	0	0	0.673 (0.495 - 0.818)	0.019 (0.001 - 0.098)	0.302 (0.163 - 0.473)	1	0
	Chisamba	1	30	0	0	0.041 (0.005 - 0.127)	0.959 (0.873 - 0.995)	0	0	0.738 (0.569 - 0.861)	1.620e-05 (2.986e-08 - 0.015)	0.262 (0.139 - 0.43)	1	0
	Chitambo	1	24	0	0	0.015 (0.002 - 0.048)	0.985 (0.952 - 0.998)	0	0	0.852 (0.772 - 0.912)	0.025 (0.006 - 0.071)	0.119 (0.065 - 0.192)	1	0
	Kabwe	1	29	0	0	0.037 (0.007 - 0.093)	0.963 (0.907 - 0.993)	0	0	0.876 (0.778 - 0.943)	0.03 (0.004 - 0.094)	0.088 (0.037 - 0.172)	1	0
	Luano	1	27	0	0	0.037 (0.015 - 0.08)	0.963 (0.92 - 0.985)	0	0	0.877 (0.804 - 0.929)	0.04 (0.015 - 0.086)	0.081 (0.04 - 0.143)	1	0
	Mkushi	1	26	0	0	0.013 (0 - 0.068)	0.987 (0.932 - 1)	0	0	0.931 (0.846 - 0.979)	0.012 (0 - 0.061)	0.053 (0.013 - 0.13)	1	0

	Serenje	1	73	0	0	0.075 (0.038 - 0.125)	0.918 (0.866 - 0.957)	0	0.005 (0 - 0.025)	0.835 (0.755 - 0.891)	3.978e-08 (1.349e-12 - 0.003)	0.165 (0.108 - 0.245)	1 (1 - 1)	4.993e-08 (3.822e-08 - 5.313e-08)
Luapula	Chembe	1	75	6.170e-12 (1.062e-12 - 0.005)	0	0.047 (0.008 - 0.167)	0.952 (0.833 - 0.992)	0	0	0.793 (0.65 - 0.91)	1.270e-05 (2.823e-08 - 0.019)	0.205 (0.09 - 0.349)	1	0
	Chifunabuli	1	26	0	0.019 (0.003 - 0.062)	0.008 (0 - 0.044)	0.969 (0.916 - 0.993)	1.455e-05 (3.950e-08 - 0.007)	0	0.824 (0.731 - 0.895)	0	0.176 (0.105 - 0.269)	1 (1 - 1)	4.330e-08 (3.003e-08 - 5.856e-08)
	Chipili	1	30	0	0	0.036 (0.016 - 0.069)	0.964 (0.931 - 0.984)	0	0	0.911 (0.861 - 0.944)	3.036e-12 (1.028e-12 - 0.001)	0.089 (0.056 - 0.139)	1	0
	Lunga	1	46	0	0.051 (0.024 - 0.092)	0.025 (0.007 - 0.059)	0.923 (0.874 - 0.958)	0	0	0.85 (0.794 - 0.903)	0.004 (4.105e-05 - 0.023)	0.143 (0.093 - 0.2)	1	0
	Milenge	1	65	0	0	0	0	0	0	0.96 (0.877 - 0.993)	0.04 (0.007 - 0.123)	2.958e-12 (1.021e-12 - 0.003)	1	0
	Mwansa bombwe	1	37	0	1.389e-05 (3.100e-08 - 0.024)	0.085 (0.016 - 0.233)	0.912 (0.764 - 0.984)	0	0	0.948 (0.808 - 0.996)	0	0.052 (0.004 - 0.192)	1	0
	Mwense	1	29	0	0	0.059 (0.012 - 0.174)	0.941 (0.826 - 0.988)	0	0	0.918 (0.803 - 0.974)	0	0.082 (0.026 - 0.197)	1	0
	Nchelenge	1	17	0	0	0.021 (0.001 - 0.114)	0.979 (0.886 - 0.999)	0	0	0.889 (0.734 - 0.966)	0	0.111 (0.034 - 0.266)	1	0
	Samfya	1	25	0	0	0.091 (0.027 - 0.204)	0.909 (0.796 - 0.973)	0	0	0.878 (0.747 - 0.957)	0	0.122 (0.043 - 0.253)	1	0
Northern	Luwingu	1	27	0.004 (0 - 0.023)	1.175e-05 (3.322e-08 - 0.006)	0.058 (0.029 - 0.105)	0.934 (0.886 - 0.966)	0	0	0.88 (0.81 - 0.928)	0.004 (0 - 0.02)	0.114 (0.068 - 0.183)	1	0
	Mbala	1	28	1.933e-12 (1.002e-12 - 3.448e-11)	0.01 (0.001 - 0.02)	0.09 (0.071 - 0.13)	0.898 (0.865 - 0.913)	0	0	0.773 (0.697 - 0.832)	0.017 (0.005 - 0.041)	0.208 (0.149 - 0.283)	1	0
	Mporokoso	1	65	1.696e-05 (2.789e-08 - 0.007)	1.937e-12 (1.008e-12 - 3.412e-11)	0.172 (0.089 - 0.296)	0.827 (0.704 - 0.911)	0	0	0.743 (0.606 - 0.851)	0.009 (0 - 0.043)	0.246 (0.139 - 0.378)	1	0
	Mpulungu	1	26	0	0	0.118 (0.06 - 0.198)	0.882 (0.802 - 0.94)	0	0	0.793 (0.678 - 0.875)	0.016 (0.002 - 0.054)	0.186 (0.108 - 0.303)	1	0
	Mungwi	1	29	0	0	0.178 (0.107 - 0.271)	0.822 (0.729 - 0.893)	0	0	0.763 (0.659 - 0.853)	0	0.237 (0.147 - 0.341)	1	0
	Senga	1	29	0.007 (0.001 - 0.02)	0.002 (0 - 0.014)	0.089 (0.053 - 0.137)	0.9 (0.848 - 0.937)	0	0	0.85 (0.784 - 0.897)	0	0.15 (0.103 - 0.216)	1	0

Muchinga	Kanchibiya	1	47	0.004 (0 - 0.023)	0	0.038 (0.014 - 0.074)	0.955 (0.916 - 0.982)	0	0	0.793 (0.707 - 0.864)	0.07 (0.037 - 0.125)	0.132 (0.079 - 0.206)	1	0
	Lavushimanda	1	75	0.021 (0.003 - 0.07)	0	0.047 (0.014 - 0.108)	0.928 (0.857 - 0.972)	0	0	0.731 (0.598 - 0.837)	0.053 (0.015 - 0.126)	0.208 (0.113 - 0.334)	1	0
	Mafinga	1	25	0	0	0.022 (0.001 - 0.127)	0.978 (0.873 - 0.999)	0	0	0.836 (0.672 - 0.941)	0.058 (0.009 - 0.175)	0.095 (0.019 - 0.234)	1	0
	Nakonde	2	32	0	0	0.184 (0.084 - 0.324)	0.816 (0.676 - 0.916)	0	0	0.648 (0.548 - 0.814)	1.646e-12 (1.014e-12 - 3.116e-11)	0.352 (0.186 - 0.452)	1	0
	Shiwangandu	1	13	0.012 (0 - 0.059)	0	0.139 (0.063 - 0.267)	0.845 (0.707 - 0.927)	0	0	0.785 (0.643 - 0.894)	0.011 (0 - 0.063)	0.196 (0.093 - 0.342)	1	0
Eastern	Chipata	1	77	0	0	0	1	0	0	0.939 (0.841 - 0.986)	0.04 (0.006 - 0.123)	0.014 (0.001 - 0.07)	1	0
	Katete	1	27	0	4.489e-06 (1.178e-12 - 0.004)	0.122 (0.071 - 0.198)	0.878 (0.802 - 0.929)	0	0	0.772 (0.683 - 0.849)	0.055 (0.022 - 0.104)	0.17 (0.101 - 0.246)	1	0
	Nyimba	1	29	0	0	0.014 (0.001 - 0.077)	0.986 (0.923 - 0.999)	0	0	0.885 (0.758 - 0.96)	0.067 (0.016 - 0.175)	0.037 (0.006 - 0.126)	1	0
	Petauke	1	18	0	0	0.061 (0.02 - 0.139)	0.939 (0.861 - 0.98)	0	0	0.921 (0.825 - 0.972)	0	0.079 (0.028 - 0.175)	1	0
	Sinda	1	26	1.302e-05 (3.249e-08 - 0.013)	0.014 (0.001 - 0.064)	0.015 (0.001 - 0.066)	0.965 (0.895 - 0.994)	0	0	0.909 (0.808 - 0.97)	0.011 (0 - 0.07)	0.073 (0.022 - 0.164)	1	0
	Vubwi	1	28	1.821e-12 (1.013e-12 - 3.844e-11)	0	0.039 (0.019 - 0.077)	0.961 (0.923 - 0.981)	0	0	0.909 (0.856 - 0.949)	0	0.091 (0.051 - 0.144)	1	0

[a]: Number of health facilities within district. [b]: number of samples used to estimate allele frequencies

Table S12: Proportion of samples carrying a *dhfr* haplotype. Source data for Figure 3C.

Number of samples carrying the haplotype (% of total, *N*)

		**Country**	**Western**	**North Western**	**Southern**	**Copperbelt**	**Lusaka**	**Central**	**Luapula**	**Northern**	**Muchinga**	**Eastern**
***dhfr* haplotype**	***N***	2379	193	244	219	287	209	293	344	201	190	199
	**Classification**											
AICNI	<3 mutations	45 (1.89%)	6 (3.11%)	5 (2.05%)	15 (6.85%)	3 (1.05%)	2 (0.96%)	2 (0.68%)	4 (1.16%)	4 (1.99%)	3 (1.58%)	1 (0.5%)
ANRNI	<3 mutations	23 (0.97%)	1 (0.52%)	0	14 (6.39%)	1 (0.35%)	0	4 (1.37%)	0	2 (1%)	0	1 (0.5%)
ANCNI	<3 mutations	1 (0.04%)	0	0	0	0	0	0	0	0	1 (0.53%)	0
AICNI+ANRNI	<3 mutations	3 (0.13%)	0	0	3 (1.37%)	0	0	0	0	0	0	0
AICNI+AIRNI	Triple	117 (4.92%)	12 (6.22%)	12 (4.92%)	7 (3.2%)	12 (4.18%)	4 (1.91%)	7 (2.39%)	38 (11.05%)	12 (5.97%)	12 (6.32%)	1 (0.5%)
AICNI+AIRNI+ANRNI	Triple	16 (0.67%)	4 (2.07%)	3 (1.23%)	2 (0.91%)	0	0	1 (0.34%)	4 (1.16%)	1 (0.5%)	1 (0.53%)	0
AIRNI	Triple	2101 (88.31%)	164 (84.97%)	215 (88.11%)	175 (79.91%)	261 (90.94%)	200 (95.69%)	266 (90.78%)	282 (81.98%)	176 (87.56%)	166 (87.37%)	196 (98.49%)
AIRNI+ANRNI	Triple	67 (2.82%)	6 (3.11%)	9 (3.69%)	3 (1.37%)	9 (3.14%)	3 (1.44%)	13 (4.44%)	13 (3.78%)	6 (2.99%)	5 (2.63%)	0
AIRNI+AIRSI	Triple	1 (0.04%)	0	0	0	1 (0.35%)	0	0	0	0	0	0
AIRNL	Triple	1 (0.04%)	0	0	0	0	0	0	0	0	1 (0.53%)	0
AIRNI+AIRNL	Quadruple	1 (0.04%)	0	0	0	0	0	0	0	0	1 (0.53%)	0
Undetermined[a]	Undetermined	3 (0.13%)	0	0	0	0	0	0	3 (0.87%)	0	0	0

[a]Mixed genotype in more than 2 targets.

Table S13: Proportion of samples carrying a *dhps* haplotype. Source data for Figure 3C.

Number of samples carrying the haplotype (% of total, *N*)

dhps haplotype		Country	Western	North Western	Southern	Copperbelt	Lusaka	Central	Luapula	Northern	Muchinga	Eastern
	N	2257	183	233	200	271	206	282	323	187	179	193
	**Classification**											
ISAKAA	Wild type	44 (1.95%)	4 (2.19%)	1 (0.43%)	5 (2.5%)	3 (1.11%)	4 (1.94%)	4 (1.42%)	12 (3.72%)	6 (3.21%)	4 (2.23%)	1 (0.52%)
IAAKAA	<2 mutations	2 (0.09%)	0	0	0	1 (0.37%)	0	0	0	1 (0.53%)	0	0
IAAKAA+ISGKAA	<2 mutations	3 (0.13%)	1 (0.55%)	1 (0.43%)	0	0	0	0	1 (0.31%)	0	0	0
IAGKAA	<2 mutations	14 (0.62%)	2 (1.09%)	5 (2.15%)	0	0	3 (1.46%)	2 (0.71%)	2 (0.62%)	0	0	0
IAGKAA+ISGKAA	<2 mutations	3 (0.13%)	0	1 (0.43%)	0	0	0	1 (0.35%)	0	1 (0.53%)	0	0
ISAEAA	<2 mutations	2 (0.09%)	0	0	0	0	0	0	0	0	2 (1.12%)	0
ISAEAA+ISAKAA	<2 mutations	2 (0.09%)	1 (0.55%)	0	0	0	0	0	0	0	1 (0.56%)	0
ISAKAA+ISGKAA	<2 mutations	4 (0.18%)	1 (0.55%)	0	0	2 (0.74%)	0	0	1 (0.31%)	0	0	0
ISGKAA	<2 mutations	102 (4.52%)	12 (6.56%)	24 (10.3%)	21 (10.5%)	17 (6.27%)	9 (4.37%)	9 (3.19%)	4 (1.24%)	3 (1.6%)	2 (1.12%)	1 (0.52%)
IAGEAA+ISAEAA+ISGEAA	Double	1 (0.04%)	0	0	0	0	0	0	0	1 (0.53%)	0	0
IAGEAA+ISGEAA	Double	1 (0.04%)	1 (0.55%)	0	0	0	0	0	0	0	0	0
ISAEAA+ISGEAA	Double	9 (0.4%)	1 (0.55%)	0	0	2 (0.74%)	0	3 (1.06%)	1 (0.31%)	2 (1.07%)	0	0
ISGEAA	Double	1461 (64.73%)	87 (47.54%)	85 (36.48%)	119 (59.5%)	177 (65.31%)	170 (82.52%)	215 (76.24%)	160 (49.54%)	126 (67.38%)	139 (77.65%)	183 (94.82%)
ISGEAA+ISGEAV	Double	1 (0.04%)	0	0	0	0	0	0	0	0	1 (0.56%)	0
ISGEAA+ISGKAA	Double	222 (9.84%)	34 (18.58%)	65 (27.9%)	32 (16%)	22 (8.12%)	11 (5.34%)	10 (3.55%)	34 (10.53%)	6 (3.21%)	7 (3.91%)	1 (0.52%)
ISGEAA+ISGEAS	Triple	1 (0.04%)	0	0	0	0	0	0	1 (0.31%)	0	0	0
ISGEAA+ISGEGA	Triple	45 (1.99%)	6 (3.28%)	1 (0.43%)	2 (1%)	11 (4.06%)	0	6 (2.13%)	7 (2.17%)	11 (5.88%)	1 (0.56%)	0
ISGEAA+ISGEGA+ISGKAA	Triple	14 (0.62%)	1 (0.55%)	2 (0.86%)	3 (1.5%)	3 (1.11%)	0	1 (0.35%)	3 (0.93%)	1 (0.53%)	0	0
ISGEGA	Triple	18 (0.8%)	4 (2.19%)	1 (0.43%)	0	4 (1.48%)	1 (0.49%)	0	0	4 (2.14%)	1 (0.56%)	3 (1.55%)
ISGEGA+ISGKAA	Triple	3 (0.13%)	1 (0.55%)	1 (0.43%)	0	0	0	0	0	1 (0.53%)	0	0
Undetermined[a]	Undetermined	305 (13.51%)	27 (14.75%)	46 (19.74%)	18 (9%)	29 (10.7%)	8 (3.88%)	31 (10.99%)	97 (30.03%)	24 (12.83%)	21 (11.73%)	4 (2.07%)

[a]Mixed genotype in more than 2 targets.

Table S14: Proportion of samples carrying a *dhps-dhfr* haplotype. Source data for Figure 3C.

Number of samples carrying the haplotype (% of total, *N*)

		**Country**	**Western**	**North Western**	**Southern**	**Copperbelt**	**Lusaka**	**Central**	**Luapula**	**Northern**	**Muchinga**	**Eastern**
***dhps-dhfr* haplotype**	***N***	2251	183	233	200	271	206	282	323	187	179	193
	**Classification**											
IAGKAA-AIRNI	<5 mutations	13 (0.58%)	2 (1.09%)	5 (2.15%)	0	0	2 (0.97%)	2 (0.72%)	2 (0.62%)	0	0	0
IAGKAA-AIRNI+ ISGKAA-AIRNI	<5 mutations	3 (0.13%)	0	1 (0.43%)	0	0	0	1 (0.36%)	0	1 (0.53%)	0	0
ISAKAA-AIRNI	<5 mutations	40 (1.78%)	2 (1.09%)	1 (0.43%)	5 (2.53%)	2 (0.74%)	4 (1.94%)	4 (1.43%)	12 (3.72%)	6 (3.21%)	3 (1.68%)	1 (0.52%)
ISGEAA-AICNI	<5 mutations	33 (1.47%)	4 (2.19%)	3 (1.29%)	14 (7.07%)	2 (0.74%)	2 (0.97%)	1 (0.36%)	2 (0.62%)	3 (1.6%)	1 (0.56%)	1 (0.52%)
ISGEAA-ANRNI	<5 mutations	19 (0.84%)	0	0	11 (5.56%)	1 (0.37%)	0	4 (1.43%)	0	2 (1.07%)	0	1 (0.52%)
ISGKAA-AICNI	<5 mutations	3 (0.13%)	0	2 (0.86%)	0	0	0	1 (0.36%)	0	0	0	0
ISGKAA-AIRNI	<5 mutations	97 (4.31%)	12 (6.56%)	22 (9.44%)	20 (10.1%)	17 (6.27%)	9 (4.37%)	8 (2.87%)	3 (0.93%)	3 (1.6%)	2 (1.12%)	1 (0.52%)
IAAKAA-AIRNI	<5 mutations	2 (0.09%)	0	0	0	1 (0.37%)	0	0	0	1 (0.53%)	0	0
ISAKAA-AICNI	<5 mutations	3 (0.13%)	1 (0.55%)	0	0	1 (0.37%)	0	0	0	0	1 (0.56%)	0
ISAKAA-AIRNI+ ISGKAA-AIRNI	<5 mutations	2 (0.09%)	0	0	0	2 (0.74%)	0	0	0	0	0	0
ISAKAA-AICNI+ ISGKAA-AICNI	<5 mutations	1 (0.04%)	0	0	0	0	0	0	1 (0.31%)	0	0	0
ISGKAA-AICNI+ ISGKAA-AIRNI	<5 mutations	1 (0.04%)	0	0	0	0	0	0	1 (0.31%)	0	0	0
IAGKAA-AICNI+ IAGKAA-AIRNI	<5 mutations	1 (0.04%)	0	0	0	0	1 (0.49%)	0	0	0	0	0
ISAEAA-AIRNI	<5 mutations	2 (0.09%)	0	0	0	0	0	0	0	0	2 (1.12%)	0
ISGEAA-ANCNI	<5 mutations	1 (0.04%)	0	0	0	0	0	0	0	0	1 (0.56%)	0
ISGEGA-AICNI	<5 mutations	1 (0.04%)	0	0	0	0	0	0	0	0	1 (0.56%)	0
IAAKAA-AIRNI+ ISGKAA-AIRNI	<5 mutations	2 (0.09%)	1 (0.55%)	1 (0.43%)	0	0	0	0	0	0	0	0
ISGEAA-AICNI+ ISGEGA-AICNI	<5 mutations	1 (0.04%)	0	0	0	0	0	0	0	1 (0.53%)	0	0
ISGEAA-AICNI+ ISGEAA-ANRNI	<5 mutations	3 (0.13%)	0	0	3 (1.52%)	0	0	0	0	0	0	0
ISGKAA-ANRNI	<5 mutations	1 (0.04%)	0	0	1 (0.51%)	0	0	0	0	0	0	0
ISAEAA-AIRNI+ ISAKAA-AIRNI	<5 mutations	1 (0.04%)	1 (0.55%)	0	0	0	0	0	0	0	0	0
ISAKAA-ANRNI	<5 mutations	1 (0.04%)	1 (0.55%)	0	0	0	0	0	0	0	0	0
ISGEAA-AICNI+ ISGKAA-AICNI	<5 mutations	1 (0.04%)	1 (0.55%)	0	0	0	0	0	0	0	0	0
ISAEAA-AIRNI+ ISGEAA-AIRNI	Quintuple	8 (0.36%)	1 (0.55%)	0	0	2 (0.74%)	0	2 (0.72%)	1 (0.31%)	2 (1.07%)	0	0
ISGEAA-AICNI+	Quintuple	42 (1.87%)	5 (2.73%)	2 (0.86%)	3 (1.52%)	4 (1.48%)	2 (0.97%)	3 (1.08%)	10 (3.1%)	7 (3.74%)	5 (2.79%)	1 (0.52%)

ISGEAA-AIRNI												
ISGEAA-AIRNI	Quintuple	1320 (58.64%)	76 (41.53%)	78 (33.48%)	84 (42.42%)	166 (61.25%)	163 (79.13%)	197 (70.61%)	141 (43.65%)	110 (58.82%)	126 (70.39%)	179 (93.23%)
ISGEAA-AIRNI+ ISGEAA-ANRNI	Quintuple	28 (1.24%)	2 (1.09%)	1 (0.43%)	0	3 (1.11%)	3 (1.46%)	8 (2.87%)	4 (1.24%)	4 (2.14%)	3 (1.68%)	0
ISGEAA-AIRNI+ ISGKAA-AIRNI	Quintuple	183 (8.13%)	26 (14.21%)	54 (23.18%)	30 (15.15%)	18 (6.64%)	11 (5.34%)	9 (3.23%)	25 (7.74%)	6 (3.21%)	3 (1.68%)	1 (0.52%)
ISGEAA-AIRNI+ ISGEAA-AIRSI	Quintuple	1 (0.04%)	0	0	0	1 (0.37%)	0	0	0	0	0	0
ISGEAA-AICNI+ ISGEAA-AIRNI+ ISGEAA-ANRNI	Quintuple	6 (0.27%)	0	1 (0.43%)	2 (1.01%)	0	0	0	2 (0.62%)	0	1 (0.56%)	0
ISGEAA-AIRNI+ ISGEAV-AIRNI	Quintuple	1 (0.04%)	0	0	0	0	0	0	0	0	1 (0.56%)	0
IAGEAA-AIRNI+ ISGEAA-AIRNI	Quintuple	1 (0.04%)	1 (0.55%)	0	0	0	0	0	0	0	0	0
ISGEAA-AIRNI+ ISGEGA-AIRNI	Sextuple	39 (1.73%)	6 (3.28%)	1 (0.43%)	2 (1.01%)	8 (2.95%)	0	6 (2.15%)	7 (2.17%)	8 (4.28%)	1 (0.56%)	0
ISGEAA-AIRNI+ ISGEGA-AIRNI+ ISGKAA-AIRNI	Sextuple	12 (0.53%)	1 (0.55%)	2 (0.86%)	3 (1.52%)	1 (0.37%)	0	1 (0.36%)	3 (0.93%)	1 (0.53%)	0	0
ISGEGA-AIRNI	Sextuple	17 (0.76%)	4 (2.19%)	1 (0.43%)	0	4 (1.48%)	1 (0.49%)	0	0	4 (2.14%)	0	3 (1.56%)
ISGEAA-AIRNI+ ISGEAS-AIRNI	Sextuple	1 (0.04%)	0	0	0	0	0	0	1 (0.31%)	0	0	0
ISGEAA-AIRNI+ ISGEAA-AIRNL	Sextuple	1 (0.04%)	0	0	0	0	0	0	0	0	1 (0.56%)	0
ISGEAA-AIRNL	Sextuple	1 (0.04%)	0	0	0	0	0	0	0	0	1 (0.56%)	0
ISGEGA-AIRNI+ ISGKAA-AIRNI	Sextuple	3 (0.13%)	1 (0.55%)	1 (0.43%)	0	0	0	0	0	1 (0.53%)	0	0
Undetermined[a]	Undetermined	355 (15.77%)	35 (19.13%)	57 (24.46%)	20 (10.1%)	38 (14.02%)	8 (3.88%)	32 (11.47%)	108 (33.44%)	27 (14.44%)	26 (14.53%)	4 (2.08%)

[a]Mixed genotype in more than 2 targets.

Table S15: Proportion of samples carrying an *mdr1* haplotype.

Number of samples carrying the haplotype (% of total, *N*)

	**Country**	**Western**	**North Western**	**Southern**	**Copperbelt**	**Lusaka**	**Central**	**Luapula**	**Northern**	**Muchinga**	**Eastern**
N	2022	148	206	175	253	171	231	315	188	168	167
**Haplotype**											
NFD	364 (18%)	27 (18.24%)	31 (15.05%)	25 (14.29%)	38 (15.02%)	51 (29.82%)	45 (19.48%)	43 (13.65%)	41 (21.81%)	27 (16.07%)	36 (21.56%)
NFD+NFY	2 (0.1%)	0	0	1 (0.57%)	0	0	1 (0.43%)	0	0	0	0
NFD+NYD	692 (34.22%)	45 (30.41%)	85 (41.26%)	49 (28%)	95 (37.55%)	21 (12.28%)	55 (23.81%)	161 (51.11%)	65 (34.57%)	67 (39.88%)	49 (29.34%)
NFY	4 (0.2%)	0	0	3 (1.71%)	1 (0.4%)	0	0	0	0	0	0
NYD	944 (46.69%)	71 (47.97%)	88 (42.72%)	94 (53.71%)	118 (46.64%)	99 (57.89%)	130 (56.28%)	107 (33.97%)	82 (43.62%)	73 (43.45%)	82 (49.1%)
NYD+NYY	2 (0.1%)	1 (0.68%)	0	0	0	0	0	1 (0.32%)	0	0	0
NYY	3 (0.15%)	1 (0.68%)	0	2 (1.14%)	0	0	0	0	0	0	0
Undetermined[a]	11 (0.54%)	3 (2.03%)	2 (0.97%)	1 (0.57%)	1 (0.4%)	0	0	3 (0.95%)	0	1 (0.6%)	0

[a]Mixed genotype in more than 2 targets.

Table S16: District-level *mdr1* and *mdr2* allele frequencies.

Allele frequencies (95% confidence interval)

Province	District	***mdr1* 184**		***mdr2* 492**	
		F	Y	I	V
Western	Kalabo	0.325 (0.272 - 0.378)	0.675 (0.622 - 0.728)	0.706 (0.649 - 0.756)	0.294 (0.244 - 0.351)
	Sesheke	0.348 (0.3 - 0.399)	0.652 (0.601 - 0.7)	0.623 (0.571 - 0.676)	0.377 (0.324 - 0.429)
	Shangombo	0.305 (0.253 - 0.358)	0.695 (0.642 - 0.747)	0.783 (0.738 - 0.827)	0.217 (0.173 - 0.262)
	Sikongo	0.335 (0.293 - 0.379)	0.665 (0.621 - 0.707)	0.666 (0.62 - 0.711)	0.334 (0.289 - 0.38)
North-Western	Chavuma	0.347 (0.292 - 0.405)	0.653 (0.595 - 0.708)	0.732 (0.678 - 0.788)	0.268 (0.212 - 0.322)
	Kabompo	0.307 (0.265 - 0.355)	0.693 (0.645 - 0.735)	0.605 (0.556 - 0.659)	0.395 (0.341 - 0.444)
	Manyinga	0.345 (0.31 - 0.386)	0.655 (0.614 - 0.69)	0.668 (0.628 - 0.707)	0.332 (0.293 - 0.372)
	Mwinilunga	0.355 (0.3 - 0.41)	0.645 (0.59 - 0.7)	0.669 (0.611 - 0.719)	0.331 (0.281 - 0.389)
	Zambezi	0.368 (0.31 - 0.429)	0.632 (0.571 - 0.69)	0.77 (0.724 - 0.816)	0.23 (0.184 - 0.276)
Southern	Chikankata	0.388 (0.331 - 0.447)	0.612 (0.553 - 0.669)	0.781 (0.731 - 0.828)	0.219 (0.172 - 0.269)
	Chirundu	0.327 (0.19 - 0.483)	0.673 (0.517 - 0.81)	0.736 (0.57 - 0.86)	0.264 (0.14 - 0.43)
	Kazungula	0.281 (0.156 - 0.419)	0.719 (0.581 - 0.844)	0.723 (0.561 - 0.846)	0.277 (0.154 - 0.439)
	Pemba	0.347 (0.269 - 0.44)	0.653 (0.56 - 0.731)	0.791 (0.699 - 0.862)	0.209 (0.138 - 0.301)
	Sinazongwe	0.305 (0.224 - 0.396)	0.695 (0.604 - 0.776)	0.621 (0.523 - 0.709)	0.379 (0.291 - 0.477)
Copperbelt	Chililabombwe	0.361 (0.269 - 0.46)	0.639 (0.54 - 0.731)	0.656 (0.557 - 0.751)	0.344 (0.249 - 0.443)
	Kalulushi	0.203 (0.101 - 0.349)	0.797 (0.651 - 0.899)	0.628 (0.451 - 0.79)	0.372 (0.21 - 0.549)
	Luanshya	0.324 (0.244 - 0.407)	0.676 (0.593 - 0.756)	0.683 (0.593 - 0.762)	0.317 (0.238 - 0.407)
	Lufwanyama	0.428 (0.297 - 0.561)	0.572 (0.439 - 0.703)	0.488 (0.366 - 0.604)	0.512 (0.396 - 0.634)
	Masaiti	0.365 (0.228 - 0.533)	0.635 (0.467 - 0.772)	0.626 (0.484 - 0.762)	0.374 (0.238 - 0.516)
	Mpongwe	0.516 (0.418 - 0.616)	0.484 (0.384 - 0.582)	0.913 (0.871 - 0.948)	0.087 (0.052 - 0.129)
	Mufulira	0.038 (0.006 - 0.106)	0.962 (0.894 - 0.994)	0.618 (0.482 - 0.731)	0.382 (0.269 - 0.518)
	Ndola	0.272 (0.138 - 0.422)	0.728 (0.578 - 0.862)	0.642 (0.468 - 0.794)	0.358 (0.206 - 0.532)
Lusaka	Kafue	0.019 (0.001 - 0.078)	0.981 (0.922 - 0.999)	0.745 (0.597 - 0.859)	0.255 (0.141 - 0.403)
	Rufunsa	0.625 (0.451 - 0.798)	0.375 (0.202 - 0.549)	0.799 (0.616 - 0.921)	0.201 (0.079 - 0.384)
Central	Chibombo	0.262 (0.127 - 0.428)	0.738 (0.572 - 0.873)	0.762 (0.594 - 0.893)	0.238 (0.107 - 0.406)
	Chisamba	0.317 (0.183 - 0.501)	0.683 (0.499 - 0.817)	0.573 (0.403 - 0.738)	0.427 (0.262 - 0.597)
	Chitambo	0.349 (0.248 - 0.45)	0.651 (0.55 - 0.752)	0.723 (0.62 - 0.817)	0.277 (0.183 - 0.38)
	Kabwe	0.266 (0.144 - 0.425)	0.734 (0.575 - 0.856)	0.772 (0.631 - 0.88)	0.228 (0.12 - 0.369)
	Luano	0.396 (0.26 - 0.542)	0.604 (0.458 - 0.74)	0.626 (0.48 - 0.764)	0.374 (0.236 - 0.52)
	Mkushi	0.301 (0.179 - 0.436)	0.699 (0.564 - 0.821)	0.708 (0.565 - 0.831)	0.292 (0.169 - 0.435)
	Serenje	0.353 (0.263 - 0.447)	0.647 (0.553 - 0.737)	0.62 (0.513 - 0.716)	0.38 (0.284 - 0.487)
Luapula	Chembe	0.31 (0.17 - 0.5)	0.69 (0.5 - 0.83)	0.62 (0.446 - 0.771)	0.38 (0.229 - 0.554)
	Chifunabuli	0.312 (0.217 - 0.425)	0.688 (0.575 - 0.783)	0.766 (0.659 - 0.86)	0.234 (0.14 - 0.341)
	Chipili	0.357 (0.285 - 0.433)	0.643 (0.567 - 0.715)	0.721 (0.648 - 0.786)	0.279 (0.214 - 0.352)
	Lunga	0.27 (0.202 - 0.355)	0.73 (0.645 - 0.798)	0.609 (0.525 - 0.687)	0.391 (0.313 - 0.475)
	Milenge	0.276 (0.148 - 0.436)	0.724 (0.564 - 0.852)	0.427 (0.284 - 0.593)	0.573 (0.407 - 0.716)
	Mwansabombwe	0.372 (0.176 - 0.605)	0.628 (0.395 - 0.824)	0.718 (0.475 - 0.902)	0.282 (0.098 - 0.525)
	Mwense	0.361 (0.214 - 0.52)	0.639 (0.48 - 0.786)	0.658 (0.478 - 0.795)	0.342 (0.205 - 0.522)
	Nchelenge	0.216 (0.092 - 0.376)	0.784 (0.624 - 0.908)	0.713 (0.539 - 0.846)	0.287 (0.154 - 0.461)
	Samfya	0.29 (0.158 - 0.456)	0.71 (0.544 - 0.842)	0.718 (0.541 - 0.855)	0.282 (0.145 - 0.459)
Northern	Luwingu	0.337 (0.255 - 0.437)	0.663 (0.563 - 0.745)	0.589 (0.468 - 0.694)	0.411 (0.306 - 0.532)
	Mbala	0.3 (0.224 - 0.386)	0.7 (0.614 - 0.776)	0.688 (0.6 - 0.77)	0.312 (0.23 - 0.4)
	Mporokoso	0.435 (0.277 - 0.599)	0.565 (0.401 - 0.723)	0.645 (0.5 - 0.781)	0.355 (0.219 - 0.5)
	Mpulungu	0.249 (0.152 - 0.367)	0.751 (0.633 - 0.848)	0.699 (0.559 - 0.807)	0.301 (0.193 - 0.441)
	Mungwi	0.264 (0.169 - 0.38)	0.736 (0.62 - 0.831)	0.653 (0.532 - 0.75)	0.347 (0.25 - 0.468)
	Senga	0.384 (0.292 - 0.485)	0.616 (0.515 - 0.708)	0.712 (0.621 - 0.795)	0.288 (0.205 - 0.379)
Muchinga	Kanchibiya	0.424 (0.287 - 0.561)	0.576 (0.439 - 0.713)	0.662 (0.527 - 0.774)	0.338 (0.226 - 0.473)
	Lavushimanda	0.336 (0.208 - 0.502)	0.664 (0.498 - 0.792)	0.622 (0.449 - 0.766)	0.378 (0.234 - 0.551)
	Mafinga	0.353 (0.175 - 0.582)	0.647 (0.418 - 0.825)	0.595 (0.375 - 0.795)	0.405 (0.205 - 0.625)
	Nakonde	0.373 (0.232 - 0.528)	0.627 (0.472 - 0.768)	0.736 (0.571 - 0.857)	0.264 (0.143 - 0.429)
	Shiwan-gandu	0.383 (0.236 - 0.54)	0.617 (0.46 - 0.764)	0.605 (0.437 - 0.759)	0.395 (0.241 - 0.563)
Eastern	Chipata	0.421 (0.269 - 0.58)	0.579 (0.42 - 0.731)	0.517 (0.343 - 0.686)	0.483 (0.314 - 0.657)
	Katete	0.334 (0.246 - 0.431)	0.666 (0.569 - 0.754)	0.62 (0.525 - 0.716)	0.38 (0.284 - 0.475)
	Nyimba	0.479 (0.309 - 0.633)	0.521 (0.367 - 0.691)	0.755 (0.596 - 0.872)	0.245 (0.128 - 0.404)
	Petauke	0.197 (0.098 - 0.319)	0.803 (0.681 - 0.902)	0.783 (0.654 - 0.881)	0.217 (0.119 - 0.346)
	Sinda	0.355 (0.227 - 0.501)	0.645 (0.499 - 0.773)	0.763 (0.629 - 0.867)	0.237 (0.133 - 0.371)
	Vubwi	0.354 (0.243 - 0.48)	0.646 (0.52 - 0.757)	0.756 (0.657 - 0.839)	0.244 (0.161 - 0.343)

[a]: Number of health facilities within district. [b]: number of samples used to estimate allele frequencies
